# Genome-wide epitope mapping across multiple host species reveals significant diversity in antibody responses to *Coxiella burnetii* vaccination and infection

**DOI:** 10.3389/fimmu.2023.1257722

**Published:** 2023-10-26

**Authors:** Emil Bach, Stephen F. Fitzgerald, Sarah E. Williams-MacDonald, Mairi Mitchell, William T. Golde, David Longbottom, Alasdair J. Nisbet, Annemieke Dinkla, Eric Sullivan, Richard S. Pinapati, John C. Tan, Leo A. B. Joosten, Hendrik-Jan Roest, Thomas Østerbye, Ad P. Koets, Søren Buus, Tom N. McNeilly

**Affiliations:** ^1^ Department of Immunology & Microbiology, University of Copenhagen, Copenhagen, Denmark; ^2^ Moredun Research Institute, Penicuik, United Kingdom; ^3^ Department of Bacteriology, Host-Pathogen Interaction and Diagnostic Development, Wageningen Bioveterinary Research, Lelystad, Netherlands; ^4^ Nimble Therapeutics, Inc., Madison, WI, United States; ^5^ Department of Internal Medicine, Radboud University Medical Center, Nijmegen, Netherlands; ^6^ Ministry of Agriculture, Nature and Food Quality, Den Haag, Netherlands; ^7^ Department of Infection and Immunology, Faculty of Veterinary Medicine, Utrecht University, Utrecht, Netherlands

**Keywords:** *Coxiella burnetii*, Q fever, peptide microarray, B cell epitope mapping, vaccination, serodiagnostic

## Abstract

*Coxiella burnetii* is an important zoonotic bacterial pathogen of global importance, causing the disease Q fever in a wide range of animal hosts. Ruminant livestock, in particular sheep and goats, are considered the main reservoir of human infection. Vaccination is a key control measure, and two commercial vaccines based on formalin-inactivated *C. burnetii* bacterins are currently available for use in livestock and humans. However, their deployment is limited due to significant reactogenicity in individuals previously sensitized to *C. burnetii* antigens. Furthermore, these vaccines interfere with available serodiagnostic tests which are also based on *C. burnetii* bacterin antigens. Defined subunit antigen vaccines offer significant advantages, as they can be engineered to reduce reactogenicity and co-designed with serodiagnostic tests to allow discrimination between vaccinated and infected individuals. This study aimed to investigate the diversity of antibody responses to *C. burnetii* vaccination and/or infection in cattle, goats, humans, and sheep through genome-wide linear epitope mapping to identify candidate vaccine and diagnostic antigens within the predicted bacterial proteome. Using high-density peptide microarrays, we analyzed the seroreactivity in 156 serum samples from vaccinated and infected individuals to peptides derived from 2,092 open-reading frames in the *C. burnetii* genome. We found significant diversity in the antibody responses within and between species and across different types of *C. burnetii* exposure. Through the implementation of three different vaccine candidate selection methods, we identified 493 candidate protein antigens for protein subunit vaccine design or serodiagnostic evaluation, of which 65 have been previously described. This is the first study to investigate multi-species seroreactivity against the entire *C. burnetii* proteome presented as overlapping linear peptides and provides the basis for the selection of antigen targets for next-generation Q fever vaccines and diagnostic tests.

## 1 Introduction

Q fever is a highly infectious zoonotic disease caused by the obligate intracellular gram-negative bacterium *Coxiella burnetii*. The disease is globally distributed apart from in New Zealand and Antarctica (1, 2). Humans can become infected following exposure to as few as 1–10 bacteria (3, 4), mainly through the inhalation of *C. burnetii-*containing dust particles and aerosols (5). Clinical manifestations of *C. burnetii* infection in humans range from common acute transient flu-like symptoms to persistent focalized infections resulting in endocarditis, hepatitis, and myocarditis, which can be life-threatening (6, 7). In pregnant women, its infection can provoke placentitis, leading to abnormal pregnancy outcomes, including miscarriage, pre-term delivery, and low birth weight (8).

Domestic ruminants, in particular dairy cows, sheep, and goats, are the main reservoir of human infections (9–11). In ruminants, *C. burnetii* can cause abortion, stillbirth, and delivery of weak offspring (12), with these clinical signs more commonly observed in small ruminants than in cattle (13). More recently, *C. burnetii* has been associated with metritis and infertility (14). During the peri-parturient period, ruminants can shed high numbers of bacteria via multiple routes, including milk, feces, vaginal secretions, and infected placental tissue, into the environment (15, 16).

Vaccination is regarded to be the most effective way to control the transmission of *C. burnetii*, with vaccination of ruminants considered important to reduce infections in both livestock and human populations (17). Currently, there are two commercially available Q fever vaccines: Q-VAX^®^ (CSL Seqirus) licensed for use in humans in Australia only and Coxevac^®^ (CEVA) for use in cattle and goats. Both vaccines are formalin-inactivated whole cell vaccines (WCVs) generated from the virulent phase I (PhI) form of the bacteria, which contains a full-length lipopolysaccharide (LPS) (18, 19). WCVs based on the avirulent phase II (PhII) form of the bacteria, which contains a truncated form of LPS, have previously been shown to be poorly effective in goats, guinea pigs, and mice (20–22), suggesting that LPS in the virulent PhI form is the major protective antigen in WCV PhI. However, immunization with a soluble extract from an avirulent phase II *C. burnetii* strain has been shown to confer protection against *C. burnetii* challenge in mice, guinea pigs, and *Rhesus macaques* (23). The protection elicited in this study may also have been related to the CpG adjuvant used, as similar studies of subunit *C. burnetii* vaccines in mice and guinea pigs have shown that adjuvant formulation can influence the levels of protection achieved (24, 25). We recently showed that a WCV prepared from a different PhII strain formulated with a saponin-based adjuvant was highly protective in a pregnant sheep model (26). This provides evidence that protection in this key target species is not exclusively LPS-based and involves additional antigens within the bacterial proteome.

A major issue with the current PhI WCVs is that they can induce potentially severe injection site reactions due to delayed-type hypersensitivity in individuals with pre-existing immunity to the bacteria, potentially involving responses to proteins of the Dot/Icm type IVB secretion system (27, 28). Consequently, deployment of the vaccine in humans is severely limited, with the Q-VAX^®^ vaccine currently being used only in Australia for high-risk individuals such as abattoir workers and only after negative serological and intra-dermal skin test (27, 29). The situation is similar in ruminants, with PhI WCVs being associated with injection site reactions, pyrexia, and production losses (30). A further issue is that PhI WCVs are difficult to manufacture, requiring production at biosafety level 3 (BSL3).

Due to these safety and manufacturing issues, there have been considerable efforts to develop subunit vaccines to control Q fever, which avoid both the reactogenicity of current PhI WCV and BSL3 manufacture. Initial approaches included the use of subcellular fractions of *C. burnetii*, of which vaccines based on chloroform/methanol residue (CMR) preparations of the bacteria have held most promise. However, despite the initial development of CMR-based vaccines in the 1990s and the demonstration of protection and reduced reactogenicity in rodent and primate models (31–33), these vaccines have not yet reached the market. More recently, efforts have focused on the development of recombinant protein vaccines, but with limited success. The main issue with this approach is that knowledge of the most appropriate bacterial antigens to be incorporated into such vaccines is lacking.

Several approaches have been taken to identify protective vaccine antigens within the *C. burnetii* proteome to address this knowledge gap. These broadly fall into two categories based on the knowledge that protection induced by PhI WCV involves both antibody and cell-mediated immune responses (34, 35): firstly, an *in silico* prediction of major histocompatibility complex (MHC) class I and II epitopes within ORFs in the *C. burnetii* genome (36–41) and, secondly, an antibody-based screening method to identify *C. burnetii* proteins targeted by antibodies from vaccinated individuals. This latter approach has involved a wide array of methodologies including two-dimensional immunoblotting of whole-cell lysates of *C. burnetii* (42, 43), cDNA library screening (42), and the use of protein microarrays using recombinant proteins either expressed in *Escherichia coli* (44, 45) or in *in vitro* translation systems (46). These combined approaches have identified a range of different *C. burnetii* proteins targeted by WCV [reviewed in Hendrix (2012)] (47), although the results are highly variable between studies. This may reflect differences between individuals but may also reflect limitations with the technology deployed—for example, a lack of sensitivity for conventional immunoblotting techniques or an inherent bias against proteins that are more difficult to express in recombinant form in protein microarrays.

Antibody profiling using peptide arrays is becoming increasingly commonplace in the study of many diseases. Recent advances in technology allow the synthesis of high-density peptide microarrays with over 4 million pre-addressable peptides, meaning that whole organism proteomes, including the human proteome, can now be represented on arrays in overlapping peptides (48, 49). Such arrays, while only identifying linear B cell epitopes [i.e., those derived from conformations adopted by contiguous amino acids (50)] and not conformational epitopes, are particularly useful when interrogating the antibody response to complex antigen cocktails. Furthermore, the key advantages of this technology are the avoidance of technical issues relating to protein expression and the ability to interrogate arrays on multiple occasions with antibodies from many individuals, thus allowing the building of a high level of consensus in the B cell epitope repertoire within a population.

In this study, we developed high-density peptide microarrays representing the complete *C. burnetii* proteome using available *C. burnetii* genomic information. This was used to interrogate the antibody response to a commercial and protective PhI WCV, Coxevac^®^, in sheep and goats, and a protective PhII WCV in sheep (20, 26). In addition, the array was used to characterize the antibody response following *C. burnetii* infection in sheep, goats, cattle, and humans to understand species-specific differences in the antibody response to *C. burnetii* and identify potential antigen targets for pan-species serodiagnostic tests and protein subunit vaccines.

## 2 Materials and methods

### 2.1 Serum samples

A summary of the serum samples used for probing peptide arrays, including species of origin, Q fever vaccination status, and *C. burnetii* infection status, is provided in **Table 1**. The groups were defined as follows: NEG, unvaccinated and uninfected; POS, unvaccinated and naturally infected with *C. burnetii*; PC, post-experimental challenge with *C. burnetii*; PV1, vaccinated with PhI WCV; PV1+C, vaccinated with PhI WCV then experimentally challenged with *C. burnetii*; PV2, vaccinated with PhII WCV; PV2+C, vaccinated with PhII WCV then experimentally challenged with *C. burnetii*; and UV+C, unvaccinated and experimentally challenged with *C. burnetii*.

**Table 1:** Summary of the serum samples used for probing *C. burnetii* peptide microarrays.

	Group	*n*	Vaccination status	Challenge status	Reference
**Cattle**	NEG	10	Unvaccinated	Unchallenged	(51)
	POS	10	Unvaccinated	Natural exposure	(52)
**Goat**	NEG	10	Unvaccinated	Unchallenged	(53, 54)
	UV+C	10	Unvaccinated	Challenged with *C. burnetii* strain X09003262-001	(54)
	PV1	10	Coxevac^®^ PhI WCV	Unchallenged	(55)
**Human**	NEG	15	Unvaccinated	Unchallenged	(56)
	POS	15	Unvaccinated	Natural exposure	(56)
**Sheep**	NEG	18	Unvaccinated	Unchallenged	(26)
	PV1	20	Coxevac^®^ PhI WCV	Unchallenged	(26)
	PV1+C	6	Coxevac^®^ PhI WCV	Challenged with *C. burnetii* strain Nine Mile RSA493	(26)
	PV2	20	PhII WCV	Unchallenged	(26)
	PV2+C	6	PhII WCV	Challenged with *C. burnetii* strain Nine Mile RSA493	(26)
	UV+C	6	Unvaccinated	Challenged with *C. burnetii* strain Nine Mile RSA493	(26)

PhI WCV, C. burnetii phase I whole-cell vaccine; PhII WCV, C. burnetii phase I whole-cell vaccine.

Pre- and post-vaccination serum was obtained from goats vaccinated with PhI WCV Coxevac^®^ and sheep vaccinated with either Coxevac^®^ or a protective PhII WCV (26). For these species, the serum samples were also obtained from animals before and after experimental challenge with *C. burnetii*. Additionally, sheep serum samples were analyzed from vaccinated individuals post-*C. burnetii* challenge. Samples from humans and cattle were obtained from seropositive and seronegative individuals, as determined by ELISA (cattle) (52) or immunofluorescence assay (humans) (47), with positive humans diagnosed as having chronic Q fever according to the Dutch Consensus Guidelines (56, 57). Seropositive and seronegative cattle and human samples were therefore considered to originate from naturally *C. burnetii*-exposed/unexposed individuals, respectively. All serum samples were stored at ≤-20°C prior to use. In addition, the samples were re-evaluated for antibody reactivity to *C. burnetii* by ELISA, using an IDEXX Q-Fever antibody test (IDEXX, UK) for sheep, cattle, and goat samples and a SERION ELISA classic *Coxiella burnetii* Phase 2 IgG (Serion GmbH, Würzburg, Germany) for human samples, following the manufacturer’s instructions. This demonstrated that samples from vaccinated and/or *C. burnetii*-infected individuals had significantly increased *C. burnetii*-specific antibodies relative to the appropriate negative control samples. Details of the timing of serum sample analysis in sheep relative to vaccination and challenge and ELISA data from all samples used for probing peptide arrays are shown in **Supplementary Figure S1**.

### 2.2 Peptide array design

Peptide microarrays were designed based on predicted open-reading frames (ORFs) identified from the genome sequence of *C. burnetii* Nine-Mile strain RSA493 (accession no. NC_002971). Specifically, the RSA493 genome sequence was re-annotated on the Galaxy platform (http://usegalaxy.org) (58) using Prokka v1.14.5+galaxy0 (59), followed by manual curation to correct where possible for frame-shifts in genes through detailed comparative analyses with other close *C. burnetii* whole-genome sequences in public repositories. This also identified additional predicted ORFs unique to individual genomes. Following careful analyses of these predicted ORFs, a total of 2,092 protein sequences were identified to be included in the arrays. As internal controls, the arrays also included human herpes virus 5 with 170 proteins and the tetanus toxin protein from *Clostridium tetani*. These 2,263 protein sequences amounted to a total test set of 168,792 unique 15-amino-acid peptide sequences overlapping by four amino acids. The amino acid frequencies of the 2,263 protein sequences above generated 960 random 15-amino-acid peptide sequences with approximately the same amino acid frequencies to assess background control reactivity. *Coxiella burnetii-*derived, tetanus toxin protein, and control peptide sequences were synthesized in duplicate, totaling 328,984 peptide sequences to be synthesized on the peptide microarrays.

### 2.3 Peptide microarray manufacture and probing

Microarrays were synthesized with a Nimble Therapeutics Maskless Array Synthesizer (MAS) by light-directed solid-phase peptide synthesis using an amino-functionalized support (Greiner Bio-One) coupled with a 6-aminohexanoic acid linker and amino acid derivatives carrying a photosensitive 2-(2-nitrophenyl) propyloxycarbonyl (NPPOC) protection group (Orgentis Chemicals). Amino acids (final concentration, 20 mM) were pre-mixed for 10 min in N,N-dimethylformamide (Sigma Aldrich) with N,N,N′,N′-tetramethyl-O-(1H-benzotriazol-1-yl)uronium-hexafluorophosphate (Protein Technologies, Inc.; final concentration, 20 mM) as an activator, 6-chloro-1-hydroxybenzotriazole (Protein Technologies, Inc.; final concentration, 20 mM) to suppress racemization, and N,N-diisopropylethylamine (Sigma Aldrich; final concentration, 31 mM) as base. Activated amino acids were then coupled to the array surface for 3 min. Following each coupling step, the microarray was washed with N-methyl-2-pyrrolidone (VWR International), and the site-specific cleavage of the NPPOC protection group was accomplished by irradiation of an image created by a Digital Micro-Mirror Device (Texas Instruments), projecting 365-nm-wavelength light. Coupling cycles were repeated to synthesize the full *in silico*-generated peptide library.

Prior to sample binding, the final removal of sidechain-protecting groups was performed in 95% trifluoroacetic acid (Sigma Aldrich), 4.5% reagent-grade water (Ricca Chemical Co.), and 0.5% triisopropylsilane (TCI Chemicals) for 30 min. The arrays were incubated 2× in methanol for 30 s and rinsed 4× with reagent-grade water. The arrays were washed for 1 min in TBST (1× TBS, 0.05% Tween-20), washed 2× for 1 min in TBS, and exposed to a final wash for 30 s in reagent-grade water.

The samples were diluted (human/sheep, 1:100; goat, 1:250; and bovine, 1:50) in binding buffer (0.01 M Tris-Cl, pH 7.4, 1% alkali-soluble casein, 0.05% Tween-20) and bound to arrays overnight at 4°C. After sample binding, the arrays were washed 3× in wash buffer (1× TBS, 0.05% Tween-20) at 10 min per wash. Primary sample binding was detected via Alexa Fluor^®^ 647 or cy5-conjugated anti-IgG secondary antibody (anti-human/goat/bovine IgG, Jackson ImmunoResearch; anti-sheep IgG, Sigma Aldrich). The secondary antibody was diluted (human, 1:10,000; goat/sheep, 1:20,000; bovine, 1:1,000) in secondary binding buffer (1× TBS, 1% alkali-soluble casein, 0.05% Tween-20). The arrays were incubated with secondary antibody for 3 h at room temperature, then washed 3× in wash buffer (10 min per wash), washed for 30 s in reagent-grade water, and then dried by spinning in a microcentrifuge equipped with an array holder. The fluorescent signal of the secondary antibody was detected by scanning with an Innopsys 910AL microarray scanner. The scanned array images were analyzed with proprietary Nimble Therapeutics software to extract the fluorescence intensity values for each peptide.

### 2.4 Prediction of protein localization and function

To identify transmembrane domains, full-length *C. burnetii* protein sequences were run through the TMHMM 2.0 algorithm (60). The output contained amino acid position intervals (domains) of predicted transmembrane helices (“TMhelix”), extracellular segments (“outer”), and intracellular segments (“inner”). The protein subcellular localizations of the full-length *C. burnetii* protein sequences were predicted using a standalone version of the bioinformatic tool PSORTb 3.0 (61) with organism type set to “-n” for gram-negative bacteria.

### 2.5 Determining antibody responses from peptide array data

Peptide antibody binding signals for each serum were loaded into the R statistical software (62) and normalized by subtracting the median signal of the 960 random peptides and then correcting the signal values less than 1 to 1. For each peptide synthesized in duplicate, the mean signal values were used. To evaluate the signal distribution of the random peptides, the empirical cumulative distribution function (ECDF) of their signals was estimated using equation [1]:


[1] $$F_{n}\left(t\right)=\frac{1}{n}\sum_{i=1}^{n}1_{\left[x_{i}\;\leq \;t\right]}$$

$F_{n}$ is the fraction of signal values less than or equal to a signal value t . $1_{ [x_{i}\;\leq \;t]}\;$ is the indicator function counting the number of signal values less than or equal to t . The probability of each *C. burnetii* peptide having a signal in the distribution of the random peptides was estimated by transforming the ECDF as seen in Equation 2]:


[2] $$F_{\;n}^{\prime }(t)=\frac{1-F_{n}(t)}{\text{max }(1-F_{n}(R))}$$

where R is the set of all the signal values measured for the random peptides and *t* is the measured signal of a *C. burnetii* bacterin vaccine strain peptide. The estimated probabilities (*p*-values) of the *C. burnetii* peptides equal to 0 were set to the smallest decimal value in *R* (2.225074e-308) (62).

The *p*-value of each individual peptide was adjusted by applying Fisher’s combined probability test on the *p*-value of the peptide and its neighboring peptides within the parent protein overlapping with a minimum of 11 amino acids. Overlapping peptides were determined by using the *IRanges* package (63). Since the position of the peptide within the parent protein affected the number of overlapping peptides, the degrees of freedom (two times *n* for Fisher’s combined probability test) also depended on the position of the peptide.

A critical value was estimated by taking the signal value of the peptide with an adjusted *p*-value closest to the significance level = 0.01. Peptides were grouped into response peaks within their parent proteins by using the *findpeaks* function in the *pracma* package (64) with the estimated critical value as the background threshold. Peptides outside of a response peak or within response peaks but with an adjusted *p*-value >0.01 were filtered from the final set of significant antibody responses.

To identify domain-level antibody responses, the amino acid positions of the protein domains predicted with TMHMM 2.0 (60) were compared to the amino acid positions of the antibody response peaks using the *findOverlaps* function in the *IRanges* package (63). An antibody response peak was determined to be overlapping a predicted domain if the top of the peak overlapped the domain with a minimum of six amino acids. The number of response peaks overlapping each domain were counted for each individual serum sample and used to calculate three different values for each species and treatment group: firstly, the response frequency of each domain was calculated as the proportion of individuals in a species and treatment group with at least one overlapping response peak; secondly, the median number of responses in each domain was calculated as the median of non-zero sums of response peaks from each individual in a species and treatment group overlapping the domain; finally, the coverage of each domain was calculated as the product of its response frequency and median number of responses in each species and treatment group.

The agreement in seroreactive protein domains between individual serum samples was calculated as the Jaccard similarity between all sample pairs with the following equation 3]:


[3] $$J(A,B)=\frac{\left|A\;\cap \;B\right|}{\left|A\;\cup \;B\right|}$$

where $J\left(A,B\right)$ is the Jaccard similarity between serum samples *A* and *B*, |A ∩ B| is the number of identical seroreactive domains between *A* and *B*, and |A ∪ B| is the number of unique seroreactive domains determined in both serum samples.

The pairwise Jaccard similarities were calculated between all serum samples and grouped into categories depending on the treatment and species of the pairs compared. The 48 paired sheep serum samples taken from the same 18 individuals were analyzed separately from the other serum samples. Multiple-comparison groupings resulted in different numbers of comparisons. A generalized linear model with a gaussian error distribution [R statistical software (62)] was used to model the Jaccard similarity dependency on one more of the comparison groupings (i.e., the additive effects and the interactions of the comparison groupings). The results, degrees of freedom, and number of comparisons in each group are summarized in **Supplementary Table S1**.

### 2.6 Vaccine candidate identification

All TMHMM2.0-predicted domains with a response in any species or treatment grouping had their coverage values grouped by species and positive treatment (UV + C, POS, PV1, PV1 + C, PV2, PV2 + C, and UV + C; see **Table 1**). Within each species and positive treatment group, the coverage values for the matching species negative group were subtracted for each domain (termed the domain coverage difference). Three methods were used to identify candidate vaccine antigens, namely:

Method 1: For each species and positive treatment group, the percentiles of the domain coverage differences were calculated at 1% steps. The percentiles were plotted in a line plot in **Supplementary Figure S2**, and a cutoff value at the 75% percentile was chosen from visual inspection of where all coverage differences rose above 0. Domains passing the threshold were compared among all species and positive treatment groups, and any domains found in all the groups were determined to be vaccine candidates.

Method 2: Coverage differences across all species and treatment groups were summed for each individual domain. The percentiles of the summed coverage values were estimated at 1% steps (**Supplementary Figure S3**). A cutoff at the 95% percentile was chosen to maximize the number of targets while ensuring a significant coverage difference. Domains with summed coverage differences above the threshold were determined to be vaccine candidates.

Method 3: Coverage values of TMHMM2.0-predicted domains with responses in the paired vaccinated sheep serum samples (PV1, PV1 + C, PV2, and PV2 + C; *n* = 36) were compared from negative treatment (NEG), through post-vaccine (PV1/2) to post-challenge (PV1/2 + C). Domains with coverage values consecutively increasing over time across the three treatments, for either of the vaccines (PV1 and PV2), were determined to be vaccine candidates.

### 2.7 Pan-species diagnostic antigen identification

To identify potential antigens targeted by the antibody response in *C. burnetii-*challenged individuals, which could be useful for pan-species serodiagnostic tests, only groups which had not received any *C. burnetii* vaccine were considered. The domain coverage differences for protein domains identified as vaccine candidates (**Section 2.6**) for *C. burnetii*-exposed groups (bovine POS, human POS, goat UV+C, and sheep UV+C) were summed, and heat-maps for the 50 domains with the highest sum coverage differences were plotted using pheatmap (65). To evaluate the reactivities of these domains in vaccinated individuals, heat-maps for the same 50 domains were also generated for groups which had received either a PV1 or PV2 vaccine.

### 2.8 Functional classification of candidate vaccine and diagnostic antigens

Functional protein classification analysis was performed using PANTHER (66). Duplicates of genes with more than one peptide hit were removed from the vaccine candidate and diagnostic antigen lists, leaving 467 and 50 unique gene identifiers, respectively. The gene lists were uploaded to PANTHER for functional classification selecting *C. burnetii* as the reference organism. Panther protein class (UV + C) was selected as the output ontology for all analyses.

## 3 Results

### 3.1 Exposure to *C. burnetii* through vaccination and/or infection results in an increased number of *C. burnetii*-derived seroreactive peptides

**Figure 1:**
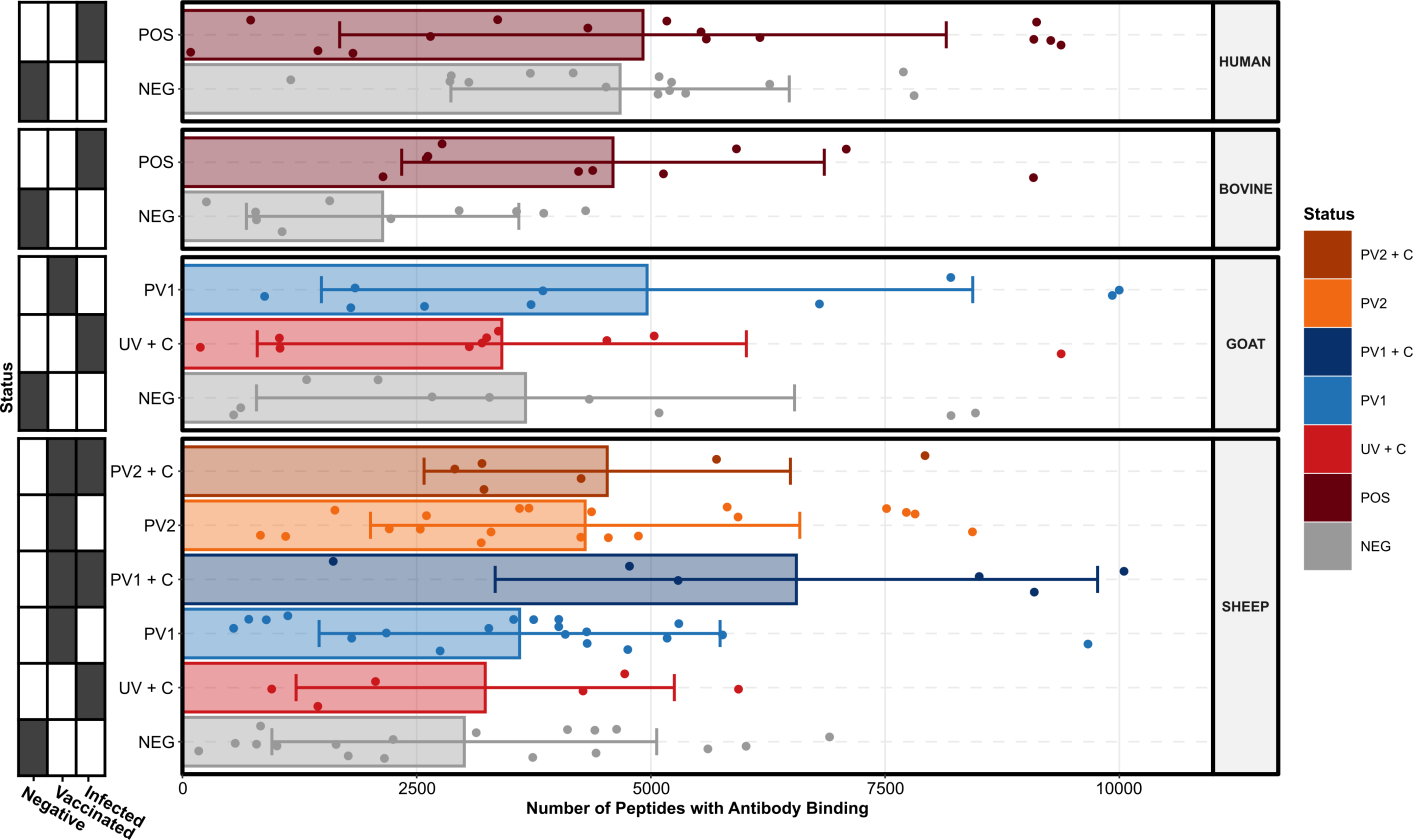
Number of *C. burnetii* peptides bound by serum antibodies stratified by species and treatment groups (*N* = 13, *n* = 156). Each point represents the peptide count in an individual serum sample, the bar heights signify the species and treatment group means, and the error bars encompass the standard deviations of each species and treatment group. The grid along the y-axis designates the exposure status of each treatment group as negative (*C. burnetii* unexposed), vaccinated (*C. burnetii* phase I/II), and/or infected (*C. burnetii* naturally infected or challenged). NEG, *C. burnetii* negative; POS, *C. burnetii* positive; PV1, post-*C. burnetii* phase I vaccination; PV2, post-*C. burnetii* phase II vaccination; UV, unvaccinated, +C, *C. burnetii* challenge.

Each peptide’s serum antibody binding degree was estimated by combining its background probability with the background probabilities of its closest overlapping neighbors, as described above. **Figure 1** summarizes the numbers of peptides with antibody binding in each serum sample across species and treatment groups. All species and treatment groups showed high variance in the numbers of antibody binding peptides. Exposure to *C. burnetii* was defined as exposure to *C. burnetii* antigens through vaccination and/or infection. Apart from the *C. burnetii* challenged goats (UV + C), all species demonstrated an increased mean number of peptides with antibody binding after exposure to *C. burnetii* through vaccination (PV1/2), natural infection (POS), or experimental challenge (UV + C) compared to their respective negative control groups (NEG). It is noteworthy that the sheep challenged with *C. burnetii* following vaccination (PV1/2 + C) had higher mean numbers of peptides with antibody binding compared to only vaccination (PV1/2), indicating a possible priming and booster effect in the antibody binding repertoire.

### 3.2 Overall agreement in seroreactive protein domains depends on species and individual

**Figure 2:**
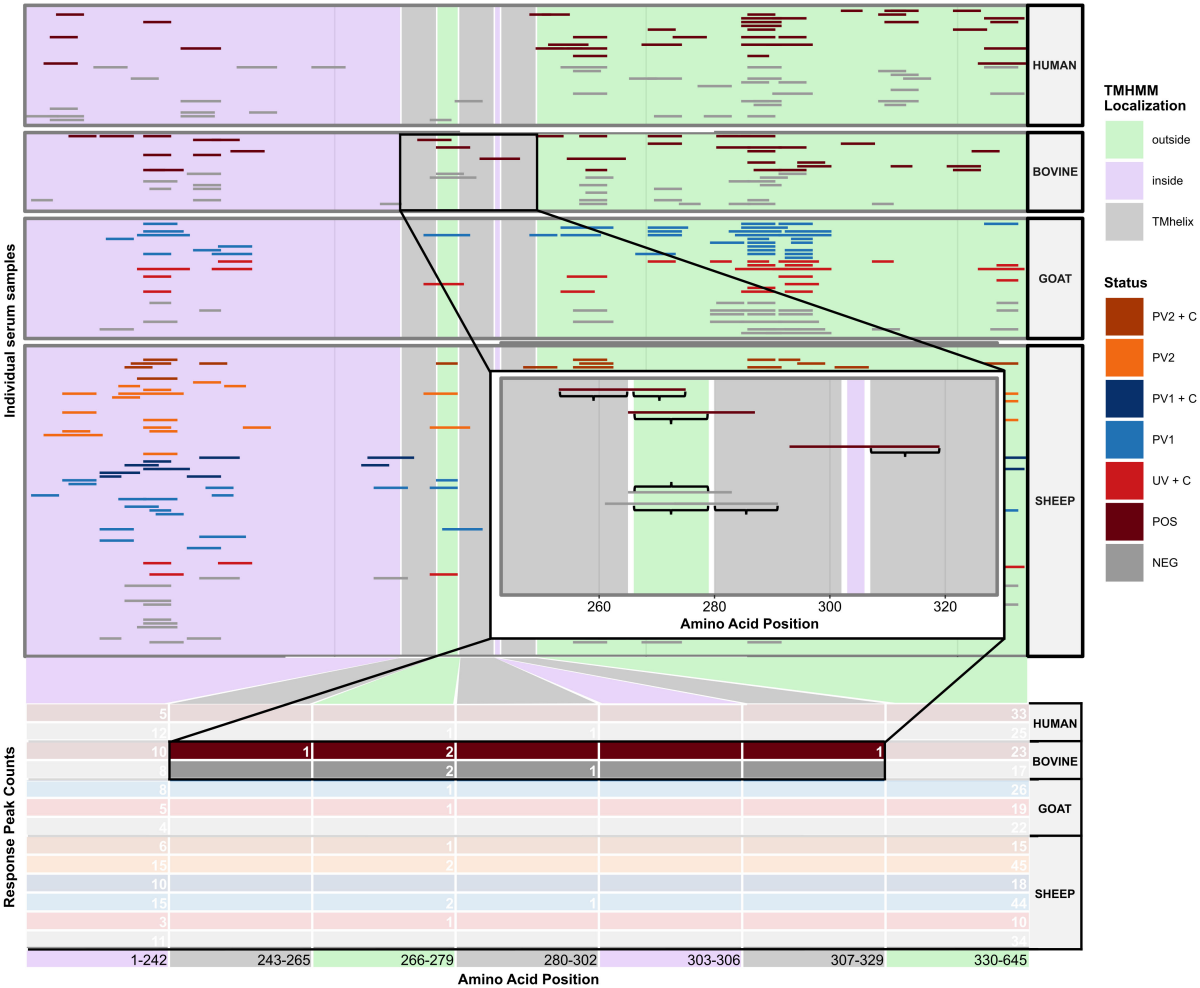
Illustration of the method used to identify domain-level antibody responses in the protein domains predicted with TMHMM2.0. The illustration uses the *C. burnetii* open reading frame *CBU_1530* as an example. On the x-axis, the amino acid positions within the protein are represented. The y-axis delineates either the individual serum samples (upper panel) or the cumulative response peak counts observed within each predicted protein domain across various species and treatment groups (lower table). The green, purple, and gray rectangles denote the predicted localizations alongside their start and end amino acid positions. The colored horizontal bars mark the start and end of antibody response peaks in individual samples. The zoomed-in plot provides a closer look at the bovine response peaks detected between CBU_1530 amino acid positions 243–329. The zoomed-in plot shows segments of response peaks encompassed by curly brackets, which indicate an overlap of six or more amino acid positions. Notably, a single response peak may overlap with multiple protein domains. NEG, *C. burnetii* negative; POS, *C. burnetii* positive; PV1, post-*C. burnetii* phase I vaccination; PV2, post-*C. burnetii* phase II vaccination; UV, unvaccinated; +C, *C. burnetii* challenge.

**Figure 3:**
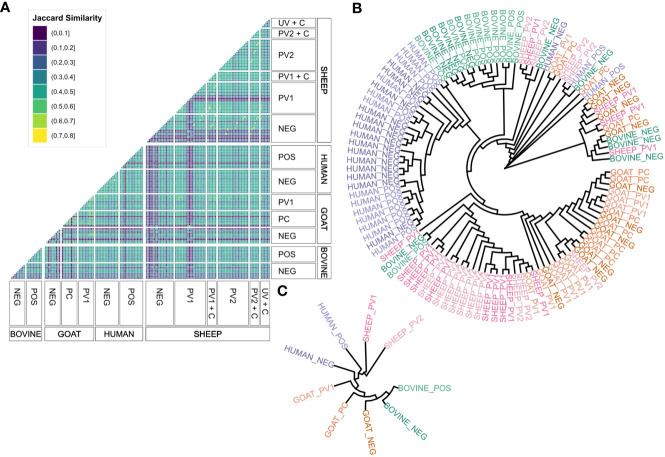
Agreement between antibody responses against TMHMM2.0-predicted domains in the *C*. *burnetii* proteins. **(A)** Faceted correlogram of left-open and right-closed intervals of the pairwise Jaccard similarities between the sets of domains with antibody responses in all serum samples (*n* = 156). **(B)** Circular dendrogram (tree) with complete-linkage clustering of the Jaccard distances (1 – Jaccard similarity) between the sets of domains with antibody responses in the unpaired serum samples (*n* = 108). The lengths of the branches are not proportional to the Jaccard distances between serum samples. **(C)** Circular dendrogram (tree) with complete-linkage clustering of the average Jaccard distances (1 – Jaccard similarity) between serum samples in the species and treatment groups with unpaired serum samples (*n* = 108, *N* = 9). The lengths of the branches are not proportional to the Jaccard distances between serum samples. NEG, *C*. *burnetii* negative; POS, *C. burnetii* positive; PV1, post-*C. burnetii* phase I vaccination; PV2, post-*C. burnetii* phase II vaccination; UV, unvaccinated; +C, *C. burnetii* challenge.

The initial goal was to examine the agreement in seroreactivity against *C. burnetii* peptides between individuals, within and between species and treatment groups, to identify selective *C. burnetii* serodiagnostic markers and protein subunit vaccine candidates. However, due to the interindividual variability in the number of antibody-reactive peptides (**Figure 1**), it proved difficult to identify peptides suitable for serodiagnostic markers or vaccine candidates. Subsequently, focus shifted to investigating seroreactivity agreement by utilizing *C. burnetii* protein domains predicted by TMHMM 2.0 and mapping the seroreactive peptides onto these domains as illustrated in **Figure 2**. The examination of Jaccard similarity between the sets of protein domains with antibody reactivity from all individual sera (**Figure 3A**) showed that the agreement between most serum samples was in the range of 20%–50%, regardless of species or treatment. However, some samples, such as the sheep negative (NEG), post-phase 1 vaccine (PV1), and *C. burnetii*-challenged goats (UV + C), showed low agreement with all other serum samples, whereas some samples in the sheep treatment groups showed high agreement (up to 80% Jaccard similarity). Complete clustering was performed on all serum samples (excluding the paired sheep samples) using the Jaccard distances (1 – Jaccard Similarity) between the individual sera to construct a dendrogram (**Figure 3B**), which indicated that sera mostly clustered within the same species regardless of *C. burnetii* exposure. However, some serum samples did occur in clusters with mixed species, so we examined the overall clustering between the species and treatment groups (**Figure 3C**), which confirmed the tendency for within-species clustering.

Clustering patterns in the unpaired serum samples (**Figure 3**) were quantified using a generalized linear model. This model compared the mean Jaccard similarities across various species and treatment categories (**Supplementary Table S1**) to evaluate their level of agreement. The model showed that all sera, on average, shared approximately 30% of their antibody-reactive protein domains, indicating a potential pan-species population of seroreactive protein domains, independent of the type of *C. burnetii* exposure. Across all species, the *C. burnetii*-exposed groups, through either vaccination or infection, had a significantly higher Jaccard similarity than the 30% intercept, whereas sera from the negative control groups had a significantly lower Jaccard similarity. As shown in **Figure 3**, sera from the same species had a significantly higher Jaccard similarity (**Supplementary Table S1**). However, within the same species, the agreement pattern reversed: Here the *C. burnetii*-exposed sera did not exhibit a significantly increased Jaccard similarity, whereas negative control sera did (**Supplementary Table S1**). Of note is that all the significant estimates were below 4%, indicating that any impact on the overall agreement was low (**Supplementary Table S1**), confirming the species-dependent agreement in seroreactivity observed in **Figure 3**. However, we did find a tendency for *C. burnetii*-exposed sera to agree on seroreactive antigens across species.

A similar generalized linear model for Jaccard similarities was used to analyze the paired sheep serum samples (**Supplementary Table S1**). Akin to the pan-species model, the intercept indicated a background of 30% Jaccard similarity, regardless of treatment or individual. Interestingly, sera from the same individuals showed the highest agreement, with 20% higher Jaccard similarity relative to the intercept. Sera from the same positive treatment groups had no increase in Jaccard similarity, but the *C. burnetii*-vaccinated and/or challenged sheep sera (PV1, PV2, PV1 + C, PV 2 + C, and UV + C) had the second highest agreement with a 10% significant increase in Jaccard similarity relative to the intercept. Conversely, the serum samples from the sheep negative control group had significantly lower Jaccard similarity than the intercept. Finally, sera from the same individuals post-*C. burnetii* vaccination and/or challenge had no increase in their Jaccard similarity. Thus, sera from the same individuals had the most antibody-reactive protein domains in common independent of the type of *C. burnetii* exposure, followed by sera from sheep with *C. burnetii* exposure through either vaccination and/or challenge.

Seroreactive protein domains in the unpaired sera agreed most within species, and for the sheep paired sera, seroreactive domains agreed most between sera sampled from the same individual. Modeling the agreement between individuals using their Jaccard similarities indicated a shared pan-species background population of seroreactive protein domains, independent of *C. burnetii* exposure status. However, agreement in seroreactivity between the positive treatment groups had a small but significant increase from this background population (**Supplementary Table S1**), indicating that some seroreactive protein domains were selective for individuals exposed to *C. burnetii* through either vaccination and/or infection.

### 3.3 Higher concordance in seroreactivity against selected protein domains in *C. burnetii*-exposed groups

**Figure 4:**
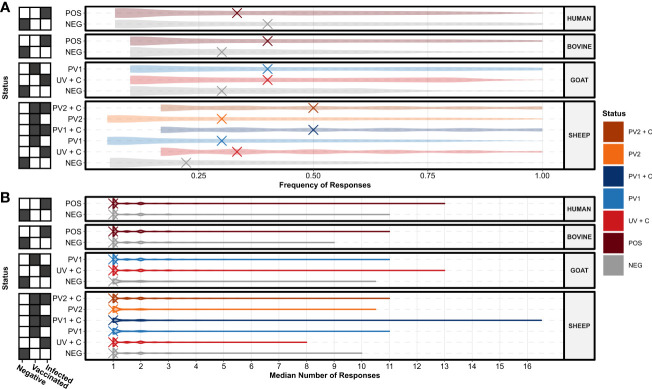
Species and treatment group antibody response levels against the TMHMM2.0-predicted domains in the *C. burnetii* proteins. **(A)** Violin plots showing the distributions of the species and treatment groups’ (*N* = 13, *n* = 156) response frequencies (fractions of individuals in a group with responses) against the predicted protein domains. The X’s denote the overall median response frequency in each species and treatment group. **(B)** Violin plots showing the distributions of the species and treatment groups’ (*N* = 13, *n* = 156) median number of responses against the predicted protein domains. The X’s denote the overall median of the median number of responses in each species and treatment group. The grids along the y-axes designate the exposure status of each treatment group as negative (*C. burnetii* unexposed), vaccinated (*C. burnetii* phase I/II), and/or infected (*C. burnetii* naturally infected or challenged). NEG, *C. burnetii* negative; POS, *C. burnetii* positive; PV1, post-*C. burnetii* phase I vaccination; PV2, post-*C. burnetii* phase II vaccination; UV, unvaccinated; +C, *C. burnetii* challenge.

To investigate the potential for seroreactive protein domains selective for *C. burnetii* vaccination and/or infection, domain seroresponse sensitivity (frequency of individuals with seroreactivity) was compared in the separate species and treatment groups (Supplementary Data 1). When comparing the seroreactive protein domain sensitivity distributions between species and treatment groups (**Figure 4A**), all groupings had at least one protein domain with an absolute sensitivity of 100%, regardless of *C. burnetii* exposure status. Apart from the sera from *C. burnetii*-exposed humans (HUMAN POS), a trend of higher median sensitivities in *C. burnetii*-exposed animals when compared to the species negative control groups was observed, indicating higher concordances in *C. burnetii* domain-directed seroreactivity in the sera from *C. burnetii*-exposed cattle (BOVINE), goats, and sheep (**Figure 4A**). However, upon examination (**Figure 4B**), this trend did not translate into the distributions of the numbers of antibody responses against these *C. burnetii* protein domains. Here the distributions of the median number of responses across the sera in each species and treatment group were found to be similarly distributed, sharing one median number of responses (**Figure 4B**). This indicated that most protein domains only had a single antibody-responsive region per individual with seroreactivity. Except for the unvaccinated sheep post-challenge sera (SHEEP UV + C), *C. burnetii-*exposed treatment groups exhibited higher maxima in the median number of responses compared to the species negative groups. (**Figure 4B**). Thus, sera from the *C. burnetii*-exposed positive treatment groups showed potential for more antibody epitopes against selected *C. burnetii* protein domains.

**Figure 5:**
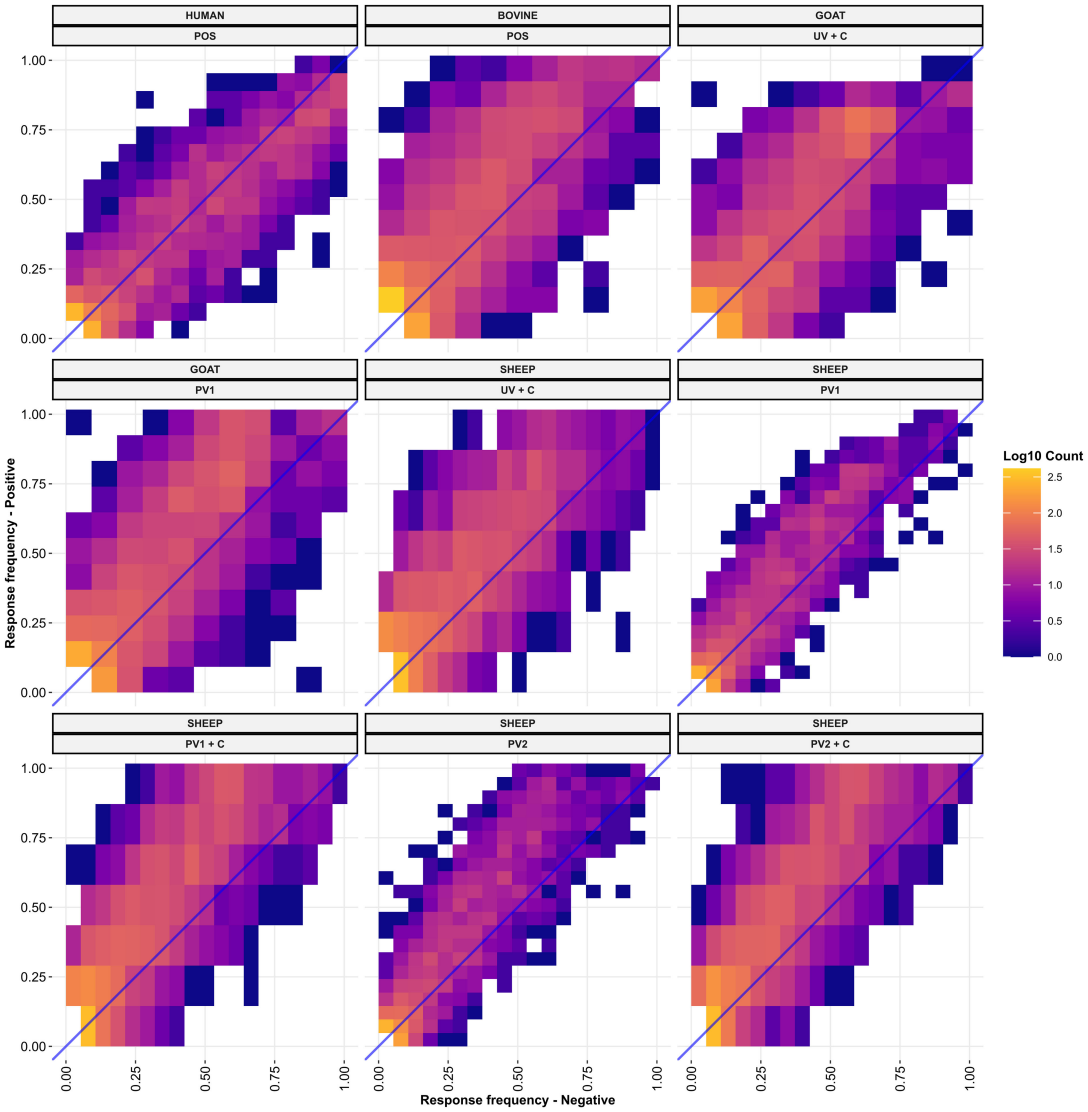
Response frequencies against each TMHMM2.0-predicted domain in the *C. burnetii* proteins compared between the positive (UV + C, POS, PV1, PV1 + C, PV2, PV2 + C, and UV + C; *N* = 9) group(s) and negative group (NEG; *N* = 4) of each species. Each panel shows a positive group (y-axis) of the indicated species *versus* the negative group of the indicated species (x-axis). The fill color of the points indicates the log base 10 to the number of predicted domains found at each point. The blue line is the diagonal identity line with a slope of 1 and an intercept at 0. NEG, *C. burnetii* negative; POS, *C. burnetii* positive; PV1, post-*C. burnetii* phase I vaccination; PV2, post-*C. burnetii* phase II vaccination; UV, unvaccinated; +C, *C. burnetii* challenge.

To understand the sensitivity and specificity of the species’ seroreactivity against individual protein domains, the frequency of antibody responders against each domain was compared between the *C. burnetii*-exposed positive treatment groups representing domain sensitivity and negative groups representing domain false positive rate (1—specificity) for each species and exposure type (**Figure 5**). When examining the distributions around the blue identity lines in the sub-plots, cattle, goats, and sheep from the *C. burnetii*-exposed treatment groups had higher numbers of seroreactive domains above the identity line, indicating an overall higher specificity in these groups (**Figure 5**). The domains were more evenly distributed around the identity line for the human samples, indicating less specificity overall (**Figure 5**). Importantly, in all species, the *C. burnetii-*exposed groups had seroreactive protein domains with 100% specificity for *C. burnetii* exposure (**Figure 5**). Notably, the goat PhI WCV Coxevac^®^ vaccination (PV1) and challenged (UV + C) treatment groups displayed selective (100% specificity) and highly sensitive seroreactivity against the same protein: CBU_0676, which was predicted as extracellular by TMHMM 2.0 (**Supplementary Data 1**). The sensitivity of this protein was 100% and 90% for the PV1 and UV + C groups, respectively (**Figure 5**; **Supplementary Data 1**). Similarly, the *C. burnetii*-exposed cattle demonstrated selective seroreactivity towards CBU_0751, another protein predicted to be extracellular by TMHMM 2.0, with a sensitivity of 80% (**Supplementary Data 1**). However, for the *C. burnetii*-exposed humans and sheep, the highest sensitivities of domains with selective seroreactivity were substantially reduced by 33% and 67%, respectively (four out of six sheep PV1 + C, **Supplementary Data 1**). Indeed the majority of the selectively seroreactive protein domains had low sensitivity, as seen in **Figure 5**. Additionally, the most sensitive protein domains were often species-specific (**Supplementary Data 1**), suggesting that different species may have distinct strategies for their humoral response against *C. burnetii*. Within species and treatment groups, we found selective seroreactivity against 703 predicted protein domains from 308 distinct *C. burnetii* proteins across the *C. burnetii*-exposed treatment groups (**Supplementary Data 1**).

### 3.4 Seroreactivity predominates in protein domains predicted as extracellular

**Figure 6:**
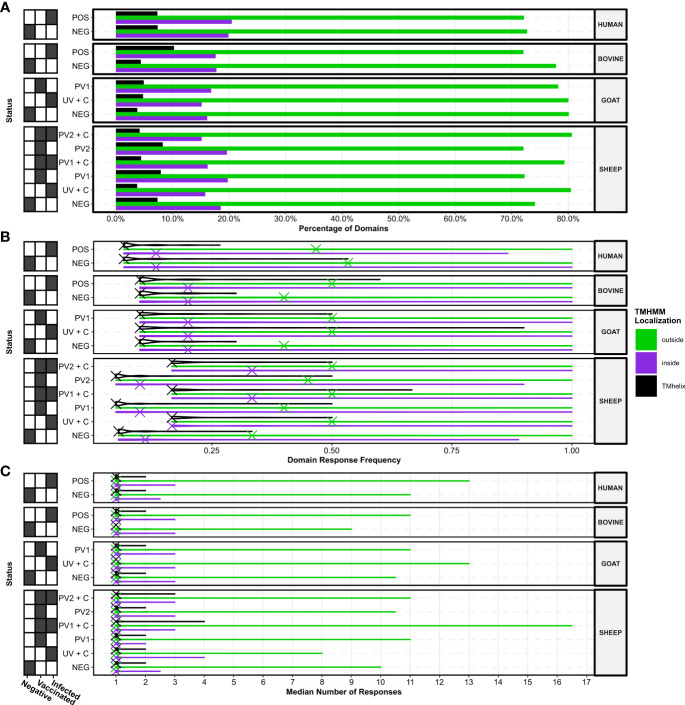
Localizations of TMHMM2.0-predicted domains in the *C*. *burnetii* proteins with responses. **(A)** Bar plots showing the percentage of domains with responses in the three different predicted localizations. **(B)** Violin plots showing the distributions of response frequencies for each species and treatment group stratified by the predicted localization of the domains. The X’s denote the overall median response frequency in each species, treatment, and localization grouping. **(C)** Violin plots showing the distributions of the median number of responses for each species and treatment group stratified by the predicted localization of the domains. The X’s denote the overall median of the median number of responses in each species, treatment, and localization grouping. The grids along the y-axes designate the exposure status of each treatment group as negative (*C. burnetii* unexposed), vaccinated (*C. burnetii* phase I/II), and/or infected (*C. burnetii* naturally infected or challenged). NEG, *C*. *burnetii* negative; POS, *C*. *burnetii* positive; PV1, post-*C. burnetii* phase I vaccination; PV2, post-*C. burnetii* phase II vaccination; UV, unvaccinated; +C, *C*. *burnetii* challenge.

By using the TMHMM 2.0 algorithm, we identified the amino acid positions and cellular localizations of protein domains in *C. burnetii* proteins. The proportions of the three different predicted localizations across the entire proteome were ~34% transmembrane helices (TMHMM 2.0: “TMhelix”), ~42% extracellular segments (TMHMM 2.0: “outside”), and ~24% intracellular segments (TMHMM 2.0: “inside”). Calculations of the proportions of the predicted localizations for protein domains with serum antibody reactivity in the species and treatment groups revealed that seroreactive domains were enriched with extracellular protein domains, appearing almost twice as often as the background proportion in the entire proteome (**Figure 6A**). In contrast, the domains with serum antibody reactivity were depleted of intracellular or transmembrane domains compared to the entire proteome (**Figure 6A**). This suggests that the humoral responses from all species, regardless of treatment, preferred potentially extracellular protein domains in the *C. burnetii* proteome.

When examining the consistency in the reactivity of protein domains between the predicted localizations across different species and treatments, the most consistently seroreactive domains were predicted as extracellular, regardless of *C. burnetii* exposure (**Figure 6B**). Furthermore, domains predicted as extracellular showed a greater overall consistency in their recognition by sera from cattle, goats, and sheep (but not humans) exposed to *C. burnetii*, as reflected in their median response frequencies, compared to their negative control groups (**Figure 6B**). The median response frequencies of protein domains predicted as intracellular were elevated in vaccinated and challenged sheep (PV1 + C and PV2 + C), and to a lesser extent in unvaccinated and challenged sheep (UV + C), compared to the sheep negative control (**Figure 6B**), but not in *C. burnetii* exposed cattle, goats, or humans (**Figure 6B**). Thus, *C. burnetii*-exposed individuals tend to display more consistency in their seroreactivity towards potentially extracellular protein domains.

The antibody response counts in protein domains between the different predicted localizations across species and treatments were analysed (**Figure 6C**). The median number of antibody responses showed no overall differences between localizations within or between species and treatments, as reflected in their median values (**Figure 6C**). However, upon examining the highest median response counts between treatments within species, we observed that the highest median response counts tended to occur in extracellular protein domains in the treatment groups exposed to *C. burnetii*. This indicates that extracellular protein domains likely harbor the largest number of potential antibody epitopes.

In conclusion, we found that extracellular protein domains were the most common target across all species, regardless of *C. burnetii* exposure. However, the concordance of seroreactivity and the quantity of potential antibody epitopes were heightened for those exposed to *C. burnetii*. This suggests that serum antibody reactivity against the linearly displayed *C. burnetii* proteome targets theoretically accessible vaccine antigens.

### 3.5 Determining potential subunit vaccine candidates across species

Our second aim was to identify antigens that could serve as effective components of a pan-species *C. burnetii* protein subunit vaccine. However, due to the absence of seroreactive protein domains with both high specificity and sensitivity across all species, we devised three alternative vaccine candidate methods: “method 1”, “method 2”, and “method 3”. These methods utilized a composite value, referred to as “coverage”, which was calculated as the product of the median antibody response count and the frequency of antibody responders (sensitivity) for each protein domain in each species and treatment group. This composite value was used to evaluate the combined seroreactivity across individuals within the same species and treatment group.

Method 1 was constructed using the difference in domain coverage between *C. burnetii*-exposed individuals (POS, PV1/2, PV1/2 + C, and UV + C) and each species’ negative control group. The vaccine candidates were selected if they had a coverage difference above the 75th percentile and were found in all species and treatment groups (**Supplementary Figure S2**). This resulted in 19 vaccine candidates (**Supplementary Table S2**).

Method 2 was based on the sum of coverage differences from all *C. burnetii*-exposed individuals across all species. The domains with coverage differences above the 95th percentile of the combined sum was chosen as vaccine candidates, resulting in 194 vaccine candidates (**Supplementary Figure S3**, **Supplementary Table S2**).

**Figure 7:**
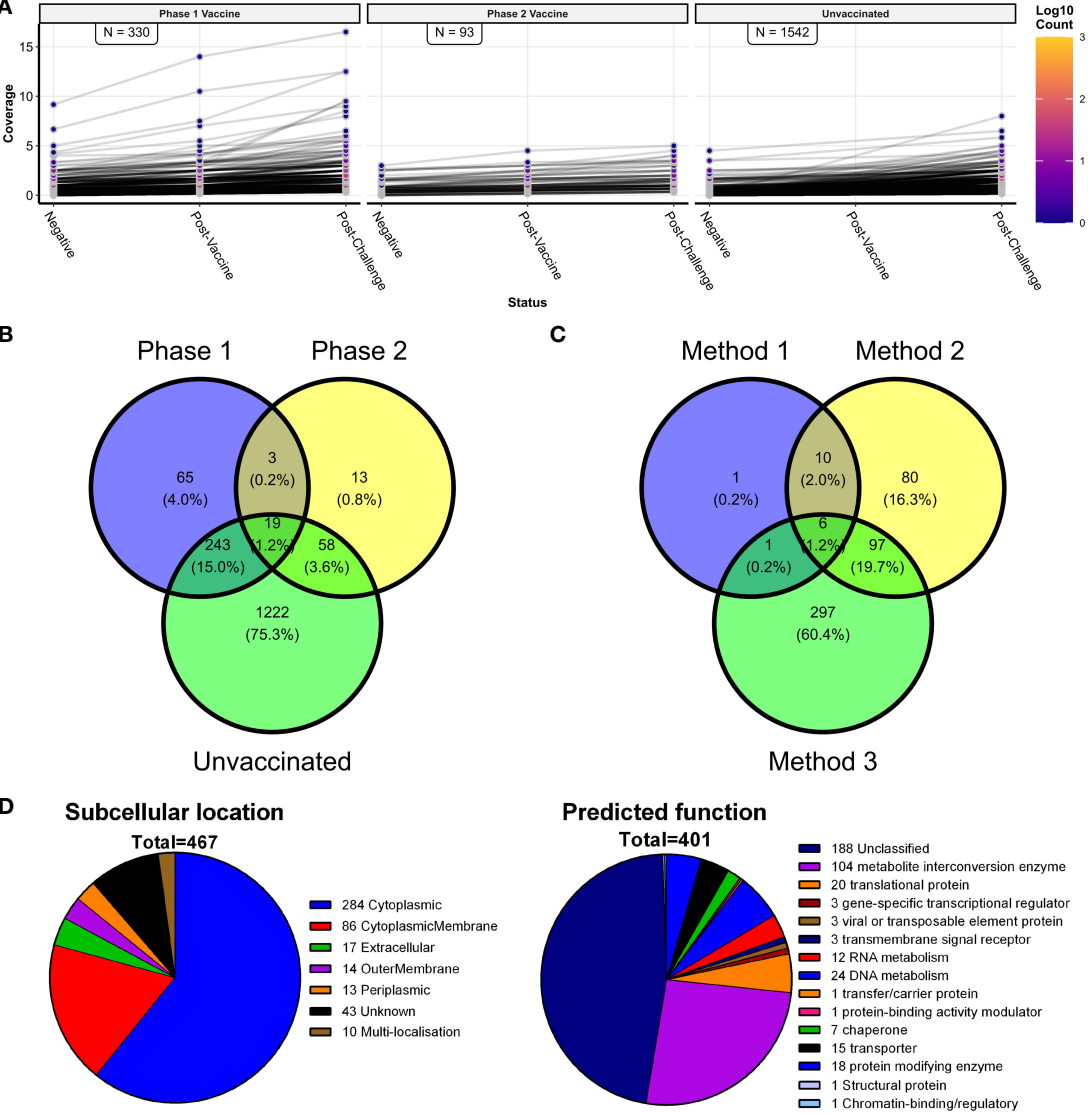
Vaccine candidate antigen identification, including the principle of method 3 and a comparison of all three vaccine methods. **(A)** Line plots of *C*. *burnetii* protein domains with consecutively increasing coverage values in the three sheep groups with paired serum samples (*n* = 48; *N* = 6). The number of domains with consecutively increasing coverage values is indicated in each facet. **(B)** Venn diagram showing the numbers and proportions of predicted domains with consecutively increasing coverage values shared between the phase 1 vaccine, phase 2 vaccine, and unvaccinated paired sheep serum samples—shown in **(A)**. **(C)** Venn diagram of the predicted protein domains found with the three vaccine target methods (methods 1, 2, and 3). **(D)** Summary of the predicted subcellular location and predicted function of the 467 identified candidate vaccine proteins represented by the 493 candidate antigen domains.

Method 3 focused on the paired sera from sheep that received either PhI or PhII of the vaccine, followed from before vaccination (NEG), after PhI/II vaccination (PV1/2), and after PhI/II vaccination and *C. burnetii* challenge (PV1/2 + C). Domain coverages were calculated for each group of six sheep at these three sample points. As potential vaccine candidates, domains that showed sequentially increasing coverage values across the three sample points were chosen (**Figure 7A**). The PhI vaccine group had more than three times the number of vaccine candidates compared to the PhII vaccine group. Additionally, the PhI vaccine candidates’ coverage values were higher than those of PhII. **Figure 7A** shows protein domains with increasing coverages in the six unvaccinated and challenged sheep (UV + C). Still these were not considered vaccine candidates since these individuals were only sampled twice, and the focus was identifying antigens targeted by the two protective vaccines. A total of 401 vaccine candidates were identified in both PhI and PhII vaccinated groups, with 22 shared between them (**Figure 7B**, **Supplementary Table S2**).

Finally, the overlap in the vaccine candidates from all three methods was examined, resulting in 493 distinct protein domains as vaccine candidates. Six vaccine candidates were common to all three methods, and all were predicted to be cytoplasmic (**Figure 7C**, **Supplementary Table S2**). The largest number of vaccine candidates was shared by method 2 with one or more of the other two vaccine candidate methods. Although method 1 had the smallest number of vaccine candidates, it shared the largest fraction (18 out of 19) with one or more of the other two vaccine candidate methods.

### 3.6 Identified *C. burnetii* vaccine candidates are associated with diverse subcellular locations and functions

An annotated list of the 493 C*. burnetii* protein domains identified as vaccine candidates using methods 1–3 is shown in **Supplementary Table S2**. These domains represented 467 individual *C. burnetii* proteins, of which 65 (13.1%) had been identified in previous antibody screening studies in mice, guinea pigs, goats, and humans. Domain localizations predicted by TMHMM 2.0 were compared to the cellular localization of the whole proteins predicted by PSORTb 3.0 in a contingency table (**Supplementary Table S3**). High levels of agreement were found between the proteins predicted as localizing to the cytoplasmic membrane, extracellular space, outer membrane, or periplasmic space by PSORTb 3.0 and the vaccine candidate protein domain localizations predicted extracellular by TMHMM 2.0. However, PSORTb 3.0 and TMHMM 2.0 showed little agreement regarding proteins predicted as cytoplasmic by PSORTb 3.0 (277/493 as cytoplasmic in total), whereas TMHMM 2.0 predicted the majority as extracellular (442/493 as outside in total). Subsequent manual curation was performed on the 467 individual candidate proteins. This assigned cellular localization to several proteins previously designated “unknown”, giving final localization: cytoplasmic (284/467), cytoplasmic membrane (86/467), extracellular (17/467), outer membrane (14/467), periplasmic (13/467) and unknown (43/467) (**Figure 7D**). A functional analysis of the 467 C*. burnetii* proteins was performed using PANTHER. This assigned a diverse range of predicted protein classes for 401/467 proteins, with the most common function being metabolite interconversion enzymes (104/401) followed by proteins involved in nucleic acid metabolism (36/401) and translational proteins (20/401) (**Figure 7D**, **Supplementary Table S2**). In total, 188/401 proteins recognized by PANTHER could not be assigned to any protein class, and PANTHER did not recognize 66/467, corresponding to uncharacterized and/or hypothetical proteins in the *C. burnetii* genome.

### 3.7 Pan-species diagnostic antigens are enriched for intracellular domains

**Figure 8:**
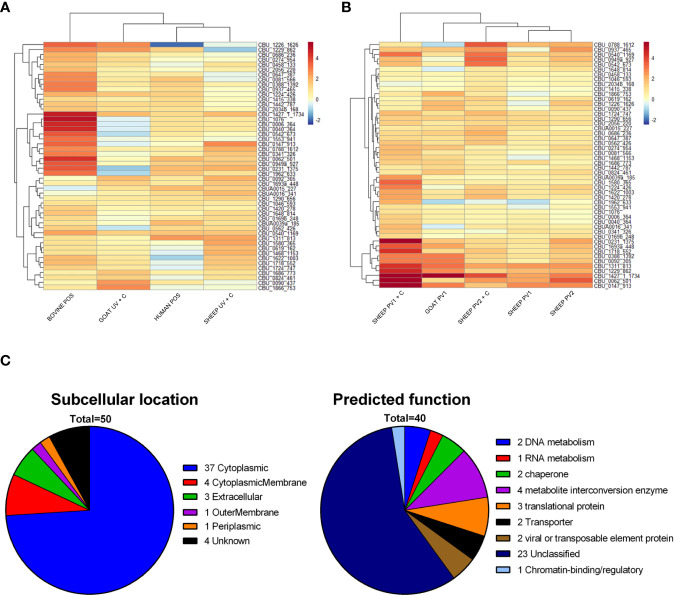
Diagnostic candidate antigen identification and predicted function and subcellular location. **(A)** Heat map of the top 50 protein domains with the highest sum coverage difference values following *C. burnetii* exposure across all species, excluding the vaccinated groups. **(B)** Heat map of the coverage difference values for the same top 50 proteins as in **(A)** for vaccinated groups. **(C)** Summary of the predicted subcellular location and predicted function of identified candidate diagnostic antigens. NEG, (*C*) *burnetii* negative; POS, *C. burnetii* positive; PV1, post-*C. burnetii* phase I vaccination; PV2, post-*C. burnetii* phase II vaccination; UV, unvaccinated; +C, *C. burnetii* challenge.

Protein domains identified by methods 1–3 with the highest summed coverage difference across all species were selected to identify antigens with pan-species serodiagnostic potential. To avoid the selection of antigens primarily recognized by vaccinated individuals, candidate antigens were considered from exposed and unvaccinated groups only (bovine POS, human POS, goat UV + C, sheep UV + C). The top 50 protein domains with the highest pan-species summed coverage difference (candidate diagnostic antigens) are shown in **Figure 8A**. Interestingly, the antibody reactivities of the 50 diagnostic candidates were highest in bovine POS samples compared to the other species. Diagnostic antigens were enriched for protein domains with a predicted intracellular localization (37/50). These were overrepresented compared to vaccine candidate antigens (74% vs. 60% predicted cytoplasmic proteins for diagnostic and vaccine antigen candidates, respectively). Protein functional analysis recognized 40/50 of the diagnostic antigens, with the majority (23/40) assigned as “unclassified” by PANTHER (**Figure 8C**). The remainder (17/40) were assigned to general metabolic protein classes. The coverage difference values of the 50 diagnostic antigens in groups receiving a vaccine showed that vaccinated sheep and goats recognized most of the candidate diagnostic antigens (**Figure 8B**).

## 4 Discussion

In this study, we investigated the serum antibody reactivity in sera from cattle, goats, humans, and sheep towards the complete predicted proteome of *C. burnetii* (predicted as 2,092 ORFs). The proteome was represented as linear and contiguous 15-amino-acid peptide sequences that were synthesized on high-density peptide microarrays. The sera were sampled from individuals with or without prior exposure to *C. burnetii* through natural infection, laboratory challenge, or vaccination using either the Coxevac^®^ PhI WCV (26, 55) or the PhII WCV (26). To our knowledge, this is the first study to perform antigen profiling of *C. burnetii* using high-density peptide microarrays. From the 156 serum samples analyzed, the antibody responses both within and between species and including *C. burnetii* exposure status were found to target diverse antigens; however, exposure to *C. burnetii* did generate a slightly more uniform seroreactivity across species. Using three separate vaccine candidate methods, 493 protein domains representing 467 potential protein subunit vaccine candidates were determined, which require further validation.

The initial examination of seroreactivity towards the *C. burnetii*-derived peptides showed that the sera from the *C. burnetii*-exposed treatment groups across all species, on average, had higher numbers of antibody-reactive peptides compared to the unexposed species-specific negative control sera, excluding the goats, which showed the opposite trend. However, antibody-reactive peptides in *C. burnetii*-negative individuals were not exclusive to the goats. Previous studies have demonstrated and confirmed that *C. burnetii* seronegative individuals could exhibit antibody reactivity towards *C. burnetii* soluble lysates (46) and/or recombinant *C. burnetii* protein antigens (44, 46, 67–70). These studies all used complete proteins as antigens. The peptide sequences used in this study, with their shorter lengths, could have greater likelihoods for sufficient homology to antigens from unrelated organisms to produce the observed cross-reactivity in the negative control groups. Furthermore, this study showed that the number of antibody-reactive peptides differed greatly between individuals within the same treatment group. The results revealed that those naturally exposed to *C. burnetii* (cattle and humans) or those challenged (goats and sheep) had the largest variation between the lowest and highest numbers of antibody-reactive peptides between individuals, with the greatest variation seen (256-fold difference) in the antibody-reactive peptides of humans naturally exposed to *C. burnetii* compared with humans who were unexposed. This variation in the number of antibody-reactive peptides could be attributed to differences in antibody titers between individuals, as reported by Beare et al. in 2008 (46) and Jeske et al. in 2021 (70), who found a difference of one to two or three orders of magnitude, respectively, between the lowest and highest titers in their human Q-fever patients. Finally, we represented *C. burnetii* protein antigens as 15 amino acid peptides based on their primary sequence, excluding both conformational epitopes and post-translationally modified epitopes found against whole antigens *in vivo*. Thus, the variations in the numbers of antibody-reactive peptides in the confirmed *C. burnetii*-exposed (i.e., vaccinated and infected) treatment groups could also be attributed to individual sera having seroreactivity targeting epitopes outside our 15-amino-acid linear peptide windows.

Due to the variable numbers of antibody-reactive peptides between individuals, the downstream analyses focused on seroreactivity at the protein antigen level. The antibody-reactive peptides were mapped to domains predicted by TMHMM2.0 (60) in their parent protein sequences. A pairwise agreement between individual sera was analysed by calculating the Jaccard similarity between their sets of seroreactive protein domains. The initial clustering based on the Jaccard distances (1—Jaccard similarity) indicated that sera from the same species had the most seroreactive protein domains in common. In quantifying the pairwise agreement with a generalized linear model, a baseline of 30% overlap in seroreactive protein domains between all individuals, independent of *C. burnetii* exposure, was observed. Individuals from the same species exhibited a slightly higher level of agreement (2%–4%); however, in the case of paired sheep samples, the sera from the same individuals shared over 50% of their seroreactive protein domains. Finally, *C. burnetii* exposure did result in significant increases in agreement (2% for unpaired sera and 10% for the paired sheep sera). Thus, the seroreactive peptides map to a background of *C. burnetii* protein domains across species and treatment groups, with some selected protein domains favored following *C. burnetii* exposure. This background of seroreactive proteins could stem from homology to other source organisms, as the study sera were taken from relatively outbred cohorts, possibly facilitating encounters with other gram-negative bacteria. Xiong et al. in 2012 (68) reported cross-reactivity against four *C. burnetii* recombinant proteins (CBU_1718, CBU_0229, CBU_1910, and CBU_0612) in 10%–30% of sera from patients with *Legionella pneumophila* infection, *Streptococcal* pneumonia, or *Rickettsial* spotted fever. Similarly, Jiao et al. in 2014 (71) observed cross-reactivity in 10%–20% of sera from mice infected with *Rickettsia heilongjiangensis* or *R. typhi* against 14 C*. burnetii* recombinant protein antigens. Furthermore, Keasey et al. in 2009 (72) found significant levels of antibody cross-reactivity among sera from rabbits inoculated with one of eight inactivated infectious gram-negative bacteria. The seroreactive antigens were classified into three categories based on their cross-reactivity levels: extensively cross-reactive, limited cross-reactive, and pathogen-selective antigens (72). Though Keasey et al. in 2009 (72) did not include *C. burnetii* in their list of pathogens, their results and the *C. burnetii* cross-reactivities reported by Xiong et al. in 2012 (68) and Jiao et al. in 2014 (71), together with observations from this study, suggest background seroreactivity against gram-negative bacteria in the negative control sera. In a recent study, Ricci et al. in 2023 used high-density peptide microarrays (produced by Nimble Therapeutics) to identify antibody epitopes against *Trypanosoma cruzi* in convalescent and healthy humans from the Americas (73). Unlike this study, their individual samples had limited shared seroreactive peptides (<30%) (73). They pooled convalescent sera based on geographical origin to screen for seroreactive peptides, subsequently analyzing targets with individual sera (73). The higher baseline at 30% overlap observed in this study may arise from testing individual sera, as pooling could dilute low-titer or low-affinity antibodies. Furthermore, Ricci et al. in 2023 employed a fixed antibody-binding signal threshold for specificity (73), while this study relied on signal distribution from randomized peptides to detect seroreactive peptides. This approach enhanced the detection sensitivity but sacrificed specificity.

Comparing the antibody reactivity between negative and exposed or vaccinated groups, a general increase in seroreactivity in *C. burnetii*-exposed or vaccinated cattle, goats, humans, and sheep, coupled with the potential for increased numbers of antibody-reactive regions within the same protein domains, was observed. Upon comparing the frequencies of antibody responses to individual protein domains between *C. burnetii*-exposed and negative controls, *C. burnetii* protein domains with selective seroreactivity for the separate *C. burnetii*-exposed treatment groups were observed. However, except for the selective and highly sensitive domain found in both the challenged and the Coxevac^®^ PhI WCV-vaccinated goats, CBU_0676, and the similarly selective and sensitive domain found in the *C. burnetii* naturally infected cattle, CBU_0751, domains with selective seroreactivity for the other *C. burnetii*-exposed treatment groups were substantially lower—peaking at 33% and 67% for the naturally infected humans and the Coxevac^®^ PhI WCV-vaccinated and *C. burnetii*-challenged sheep, respectively. Outside of the *C. burnetii-*exposed selective seroreactive domains, the sensitivities of *C. burnetii*-exposed treatment groups and species were negatively correlated, i.e., the most sensitive domains showed little specificity for the *C. burnetii-*exposed treatment groups. Thus, the seroreactive antigens of the *C. burnetii*-exposed treatment groups exhibited varying degrees of both sensitivity and specificity. A comparable observation was made by Vigil et al. in 2010 (44), who noted varying specificities and sensitivities of the seroreactivity against 64 protein antigens in their human Q-fever patients. Furthermore, the most widely reported *C. burnetii* antigen, CBU_1910 (Com1), has been found to have varying degrees of sensitivity (37.5%–55%) and specificity (71%–90%) in human Q-fever patients as summarized by Gerlach et al. in 2017 (74).

More recently, Jeske et al. in 2021 (70) determined 93% specificity and 64% sensitivity with recombinant CBU_1910 (Com1) in a human cohort of 76 C*. burnetii* seropositive and 91 negative control sera. Similarly, Stellfeld et al. in 2020 (75) found a good performance of recombinant CBU_1910 (Com1) in sera from 197 cattle, 104 goats, and 115 sheep with specificities of 70%, 77%, and 68% and sensitivities of 71%, 94%, and 85%, respectively. By comparison, this study found seroreactivity across species and treatment groups, independent of *C. burnetii* exposure, against CBU_1910, with sensitivities from 67% in the *C. burnetii*-challenged unvaccinated sheep to 100% in both the PII WCV-vaccinated and challenged sheep and the human negative control treatment groups. The results showed similar patterns for CBU_1718 (GroEL) and CBU_1290 (DnaK), which were both reported as highly performing diagnostic antigens by Jeske et al. in 2021 (70) with CBU_1718 (GroEL) and CBU_1290 (DnaK) exhibiting specificities of 69% and 77% and sensitivities of 72% and 47%, respectively. Similarly, Miller and Kersh 2020 (69) reported 90% specificity and 71.43% sensitivity for CBU_1718 (GroEL) with 24 human sera. Additionally, they found a 100% seroresponse rate in eight naturally *C. burnetii*-infected goats (69). Even though this study detected seroreactivity against these three highly rated *C. burnetii* antigens, we found limited or no selectivity compared to previous studies which used recombinant full-length antigens, highlighting how the selectivity of these antigens may depend on conformational epitopes or post-translational modifications. Of note, CBU_1718 (GroEL) and CBU_1290 (DnaK) were both selected as subunit vaccine candidates by vaccine candidate method 2 and both methods 1 and 2, respectively, indicative of higher reactivity (as measured by their coverage) compared to the negative control groups. However, the most selective antigens detected using the peptide microarrays presented low sensitivities, corresponding with the previously reported diversity in the humoral response to *C. burnetii* (44).

As noted by Gerlach et al. in 2017 (74) from the reports of seroreactivity focusing on outer membrane proteins by Hotta et al. in 2004 (76) and Papadioti et al. in 2011 (77), the theoretical best targets for an antibody-focused protein subunit vaccine should localize to the outer membrane of *C. burnetii* to neutralize cellular invasion and promote bacterial clearance. Indeed the initial analysis of protein domain localizations showed higher sensitivities (higher response frequencies in the treatment groups) and numbers of potential epitopes in domains predicted as extracellular by the TMHMM 2.0 algorithm (60). However, the assessment of the localizations of the protein antigens with PSORTb 3.0 (61) of the resulting 493 vaccine candidate domains from the three vaccine candidate methods found many discrepancies between PSORTb 3.0 and TMHMM 2.0, where PSORTb 3.0-predicted cytoplasmic proteins were predicted to have extracellular domains by TMHMM 2.0. When assessing the Gene Ontology (78, 79) molecular functions and identities of the proteins predicted cytoplasmic by PSORTb 3.0, a cytoplasmic localization was found to be the most likely. Indeed TMHMM 2.0 predicted both CBU_1718 (GroEL), a chaperonin, and CBU_1290 (DnaK), a chaperone, thus both involved in protein folding, which is expected to be associated with cytoplasmic localizations, to be localized extracellularly. Of note, CBU_1290 (DnaK) has also been predicted to be plasma-membrane-associated by UniProt (80). Furthermore, Flores-Ramirez et al. in 2014 (81) reported both CBU_1718 (GroEL) and CBU_1290 (DnaK) as having potential moonlighting activities, both involved in host cell adhesion and/or function as plasminogen receptors, thus directly aiding in host cell invasion. They reported six additional cytoplasmic proteins with potential moonlighting activities of which three, CBU_0221, CBU_0630, and CBU_1708 (81), were also detected as potential vaccine candidates by at least one of the three vaccine candidate methods used in this study. Moonlighting activities could, in part, explain the enrichment of cytoplasmic localized proteins in the vaccine candidate list produced in this study, as these would be exposed to the immune system by intact bacteria. However, cytoplasmic proteins released from lysed *C. burnetii* bacteria *in vivo* would also be exposed to the immune system and appear to be inherently immunogenic. Indeed Gerlach et al. in 2017 (74) noted an enrichment for cytoplasmic localized proteins as antigenic targets, which is consistent with our observations in this study.

Due to the observed diversity in the selective seroreactivity observed within the *C. burnetii*-exposed treatment groups (i.e., seroreactivity seen only in *C. burnetii*-exposed individuals), this study utilized the coverage as a composite value for the seroreactivity against the protein domains across individuals in the separate species and treatment groups. The rationale behind method 1 was to capture protein domains with increased antibody reactivity compared to the species negative control group shared across all *C. burnetii*-exposed treatment groups. Method 2 was a less selective approach with increased sensitivity compared to method 1, which identified protein domains with an overall increased seroreactivity across all *C. burnetii*-exposed treatment groups. Finally, method 3 utilized the uniformity in the paired sheep sera to identify protein domains that were subjected to a boost in antibody reactivity following *C. burnetii* after the challenge, thus identifying protein domains that were targeted by sheep memory B-cells activated following the *C. burnetii* challenge. Of note, the Coxevac^®^ PhI WCV-vaccinated (26) sheep treatment group produced over three times the number of vaccine candidates than the PhII WCV-vaccinated (26) sheep. Combining all three methods as shown herein yielded six protein domains targeted across different species of *C. burnetii-*exposed treatment groups while being apparent target antigens for memory B-cells in the sera from the continuously sampled sheep. These proteins were all predicted to be cytoplasmic, consistent with previous observations that *C. burnetii* cytoplasmic proteins appear to be highly antigenic (68), and one protein, CBU_0388, had previously been identified as a target of the CD8+ T cell response in *C. burnetii*-challenged mice (82).

In total, 493 vaccine candidate antigen domains, representing a total of 467 individual *C. burnetii* proteins, were identified. These proteins were predicted to be primarily (61%) cytoplasmic and to have a diverse range of functions. From this subset of proteins, 65 had been identified in previous antigen screens. Of relevance to subunit vaccine development, 31 proteins were predicted to be outer membrane or extracellular proteins and thus potential targets for antibodies with neutralization or bacterial clearance functions, such as opsonization or complement activation. Furthermore, two antigens within our candidate list, CBU_0545 and CBU_0891, have previously been shown to induce a degree of protection when formulated into a recombinant subunit vaccine with three other antigens and tested in mice (25), and a further antigen within our candidate list, CBU_0630 (Mip), has also been shown to induce protection in mice immunized with bone marrow-derived dendritic cells (BMDCs) pulsed with recombinant Mip (83). This gives some confidence that, within the candidate list produced in this study, there are antigens with the potential to induce protective immune responses. Interestingly, CBU_1910 (Com1) was also tested in both mouse vaccination studies and contributed to the protective immune response, either in combination with other antigens or following immunization with Com1-pulsed BMDCs. This antigen was not present in the vaccine candidate list produced in this study. This was surprising as Com1 is one of the most frequently identified antibody targets within the *C. burnetii* proteome (74). However, Com1 antibody reactivity has previously been identified through immunoblotting of *C. burnetii* antigen preparations or protein microarray (42, 44, 67), which may capture also conformational B cell epitopes, whereas our peptide microarrays only identify linear epitopes. Therefore, the lack of an increase in Com1 antibody reactivity in *C. burnetii*-exposed groups in this investigation could be due to Com1 primarily harboring conformational epitopes.

In addition to interrogating the data for vaccine candidates, potential serodiagnostic antigen targets were evaluated by focusing on the top 50 proteins with the highest sum coverage difference values (coverage of exposed minus negative control groups) across all species within the antigens identified through methods 1–3 but excluding groups which had been vaccinated. This approach further enriched for cytoplasmic proteins, which was again consistent with previous immune-screening studies (74), and identified nine antigens which have been identified in previous serological screens. These included CBU_1290 (DnaK), which has shown significant promise as diagnostic antigens for humans (70), and both CBU_1718 (GroEL) and CBU_0092 (YbgF), which have shown promise as diagnostic antigens in humans, sheep, cattle, and goats (69, 70, 84). Of note was that, across the four species evaluated, antibodies from naturally exposed cattle were the most reactive. Host-species-specific differences in seroreactivity proteins have previously been reported (45, 68, 71), which may be due to inherent differences in B cell receptor diversity between species. However, the variation in response may also reflect the nature of the challenge (natural exposure in cattle and humans vs. experimental *C. burnetii* challenge in sheep and goats), the duration of infection, and the strain of *C. burnetii* involved. Intriguingly, recent genotyping studies suggest that cattle are infected with distinct strains of *C. burnetii* compared to humans, goats, and sheep, which may partly explain the different seroreactivity profile in cattle (85). Comparing the reactivity of these 50 candidate diagnostic antigens in groups which have received a vaccine failed to identify any diagnostic antigen which was not also recognized post-vaccination. This reflects the initial candidate antigen identification methodology employed in this study as 25/50 (50%) of the diagnostic antigens that were identified by method 3, which focused on antibody responses in vaccinated sheep. Therefore, to ensure differentiating infected from vaccinated animals, the capability of any serodiagnostic test with these candidate antigens, it would be necessary to ensure that the antigens used for serodiagnostic assays were different from those used in prototype subunit vaccines.

## 5 Conclusion

To our knowledge, this study reports the first use of high-density peptide microarrays to profile linear B-cell epitopes in the entire proteome of *C. burnetii*. The study findings suggest that the seroreactivity against a linearly displayed *C. burnetii* proteome has marked diversity and potential cross-reactivity in *C. burnetii* seronegative individuals. This study presents a list of 493 protein domains representing 467 individual proteins as potential candidates for a protein-subunit-based *C. burnetii* vaccine. Testing the vaccine efficacy of the entire candidate list would not be feasible. Thus, the list should be scrutinized, further utilizing both predicted cellular localizations and function in the virulence of *C. burnetii* together with the potential for recombinant expression. Finally, any recombinantly expressed vaccine candidates should be tested in relevant animal models alone or in combination to determine their efficacy against the *C. burnetii* challenge.

## Data availability statement

The datasets presented in this study can be found in online repositories. The name of the repository/repositories and accession number(s) can be found below: https://datadryad.org/stash, https://doi.org/10.5061/dryad.63xsj3v44.

## Ethics statement

The studies involving humans were approved by the Medical Ethics Committee Arnhem-Nijmegen (NL35784.091.11), Radboud University Medical Center, Nijmegen, the Netherlands. The studies were conducted in accordance with the local legislation and institutional requirements. The participants provided their written informed consent to participate in this study. The animal study was approved by the Moredun Animal Welfare and Ethical Review Body (E34/18), Moredun Research Institute, Pentlands Science Park, Midlothian, EH26 0PZ UK; Utrecht University Animal Experiments Committee (2014.II.11.058; DEC2014.II.11.085; 0202.0806), Utrecht, The Netherlands; and the Wageningen Bioveterinary Research Animal Experiments Committee (AVD401002016580; 299-47053-07/99-01; 821-47302-00/01-01; 2009082.c, 2009079.a; 2010098.d; 2011111.c), Lelystad, The Netherlands. The study was conducted in accordance with the local legislation and institutional requirements.

## Author contributions

EB: Data curation, Formal Analysis, Investigation, Methodology, Writing – original draft. SF: Formal Analysis, Investigation, Methodology, Writing – original draft. SW-M: Investigation, Methodology, Writing – review & editing. MM: Investigation, Methodology, Writing – review & editing. WG: Conceptualization, Funding acquisition, Writing – review & editing. DL: Conceptualization, Funding acquisition, Methodology, Writing – review & editing. AN: Conceptualization, Funding acquisition, Writing – review & editing. AD: Writing – review & editing. ES: Formal Analysis, Methodology, Writing – review & editing. RP: Methodology, Writing – review & editing. JT: Conceptualization, Formal Analysis, Methodology, Writing – review & editing. LJ: Resources, Writing – review & editing. H-JR: Conceptualization, Funding acquisition, Writing – review & editing. TO: Formal Analysis, Writing – review & editing. AK: Conceptualization, Funding acquisition, Investigation, Resources, Writing – review & editing. SB: Conceptualization, Formal Analysis, Funding acquisition, Investigation, Writing – review & editing. TM: Conceptualization, Funding acquisition, Investigation, Methodology, Project administration, Supervision, Writing – original draft.

## Supplementary material

Supplementary FIGURE S1
*C. burnetii*-specific antibody responses in serum samples used to probe peptide arrays. **(A)** Serological responses following vaccination and/or *C. burnetii* challenge in sheep, indicating the time-points selected for peptide array analysis (26). The sheep were either vaccinated twice with a phase I *C. burnetii* bacterin vaccine (Coxevac^®^, PV1) or a phase II *C. burnetii* bacterin vaccine (PV2) or left unvaccinated. The timing of the first and second vaccinations are indicated by “V1” and “V2”, respectively. The ewes were mated on day 49 (indicated by M) and subsequently challenged on day 151 with *C. burnetii* Nine-Mile strain RSA493 (indicated by “C”). The levels of *C. burnetii*-specific antibodies within serum samples collected throughout the study were quantified by ELISA (IDEXX Q-Fever antibody test; IDEXX, UK) and presented as mean percentage positive values (PP) ± standard error of the mean (SEM). Serum samples from day 0 (NEG time-point), day 35 (PV1/PV2 time-points), and day 175 (PV1+C/PV2+C/UV+C time-points) were selected for the peptide microarray analysis. L, lambing. **(B)** Levels of *C. burnetii-*specific antibodies in humans diagnosed with chronic Q fever and uninfected controls (56) as determined by ELISA (SERION ELISA classic *Coxiella burnetii* phase 2 IgG, Serion GmbH, Würzburg, Germany). Data is presented as activity units per milliliter of serum according to the manufacturer’s instructions and shows significantly higher *C. burnetii*-specific antibody levels in the infected group compared to the negative control group (*P*< 0.0001, Mann–Whitney *U*-test). Data is presented as median values ± interquartile range. **(C)** Levels of *C. burnetii-*specific antibodies in cattle naturally infected with *C. burnetii* and uninfected controls (51, 52) as determined by ELISA (IDEXX Q-Fever antibody test; IDEXX, UK). Data is presented as mean PP values ± SEM and shows significantly higher *C. burnetii*-specific antibody levels in the infected group compared to the negative control group (*P*< 0.0001, Mann–Whitney *U*-test). **(D)** Levels of *C. burnetii-*specific antibodies in goats either experimentally infected with *C. burnetii*, vaccinated with Coxevac^®^, or uninfected controls (53–55) as determined by ELISA (IDEXX Q-Fever antibody test; IDEXX, UK). Data is presented as mean PP values ± SEM and shows significantly higher *C. burnetii*-specific antibody levels in the infected group (*P<* 0.01) and the vaccinated group (*P*< 0.0001) compared to the negative control group (one-way ANOVA followed by Tukey’s multiple-comparisons test). NEG, *C. burnetii* negative; POS, naturally infected with *C. burnetii*; PV1, post-*C. burnetii* phase I vaccination; PV2, post-*C. burnetii* phase II vaccination; UV, unvaccinated; +C, experimental *C. burnetii* challenge.

Supplementary FIGURE S2 Method 1 percentiles. Line plots showing the percentiles of differences in coverage values between the positive and negative groups within each species. The percentile values were calculated from 0% to 100% at 1% steps. Coverage is the product between a domain’s response frequency and median response count. POS, *C. burnetii* positive; PV1, post-*C. burnetii* phase I vaccination; PV2, post-*C. burnetii* phase II vaccination; UV, unvaccinated; +C, *C. burnetii* challenge.

Supplementary FIGURE S3 Method 2 percentiles. Line plot showing the percentiles of the summed differences in coverage values between the positive and negative groups within each species. The percentile values were calculated from 0% to 100% at 1% steps. Coverage is the product between a domain’s response frequency and the median response count.
