# Supplementary figures and images for: Genome-wide epitope mapping across multiple host species reveals significant diversity in antibody responses to Coxiella burnetii vaccination and infection
Source: Front Immunol. 2023 Oct 26;14:1257722. doi: 10.3389/fimmu.2023.1257722 (PMC10637584; doi:10.3389/fimmu.2023.1257722)

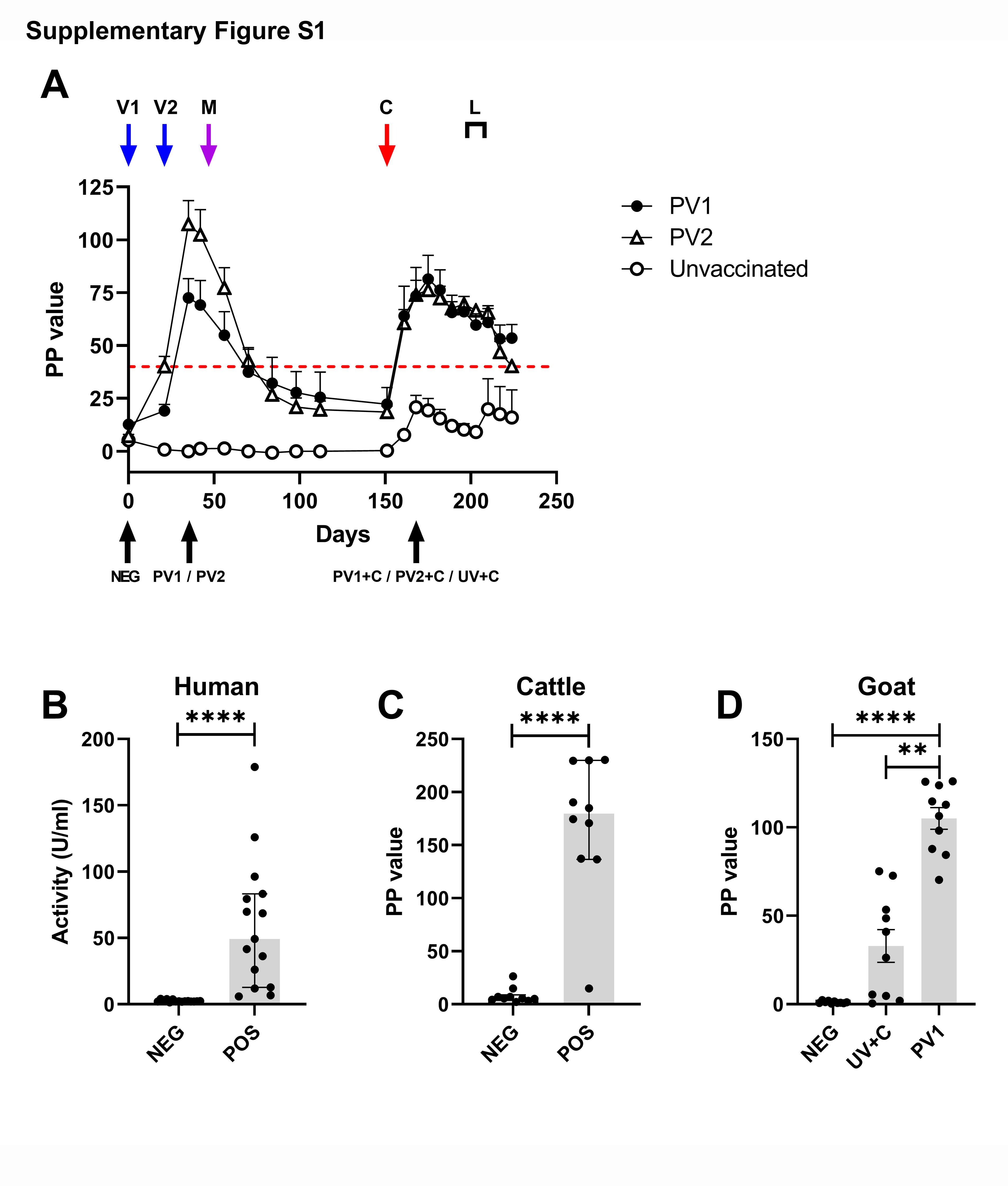

Supplement: Supplementary FIGURE S1 — C. burnetii-specific antibody responses in serum samples used to probe peptide arrays. (A) Serological responses following vaccination and/or C. burnetii challenge in sheep, indicating the time-points selected for peptide array analysis (26). The sheep were either vaccinated twice with a phase I C. burnetii bacterin vaccine (Coxevac®, PV1) or a phase II C. burnetii bacterin vaccine (PV2) or left unvaccinated. The timing of the first and second vaccinations are indicated by “V1” and “V2”, respectively. The ewes were mated on day 49 (indicated by M) and subsequently challenged on day 151 with C. burnetii Nine-Mile strain RSA493 (indicated by “C”). The levels of C. burnetii-specific antibodies within serum samples collected throughout the study were quantified by ELISA (IDEXX Q-Fever antibody test; IDEXX, UK) and presented as mean percentage positive values (PP) ± standard error of the mean (SEM). Serum samples from day 0 (NEG time-point), day 35 (PV1/PV2 time-points), and day 175 (PV1+C/PV2+C/UV+C time-points) were selected for the peptide microarray analysis. L, lambing. (B) Levels of C. burnetii-specific antibodies in humans diagnosed with chronic Q fever and uninfected controls (56) as determined by ELISA (SERION ELISA classic Coxiella burnetii phase 2 IgG, Serion GmbH, Würzburg, Germany). Data is presented as activity units per milliliter of serum according to the manufacturer’s instructions and shows significantly higher C. burnetii-specific antibody levels in the infected group compared to the negative control group (P< 0.0001, Mann–Whitney U-test). Data is presented as median values ± interquartile range. (C) Levels of C. burnetii-specific antibodies in cattle naturally infected with C. burnetii and uninfected controls (51, 52) as determined by ELISA (IDEXX Q-Fever antibody test; IDEXX, UK). Data is presented as mean PP values ± SEM and shows significantly higher C. burnetii-specific antibody levels in the infected group compared to the negative control group ( [file Image_1.tif]

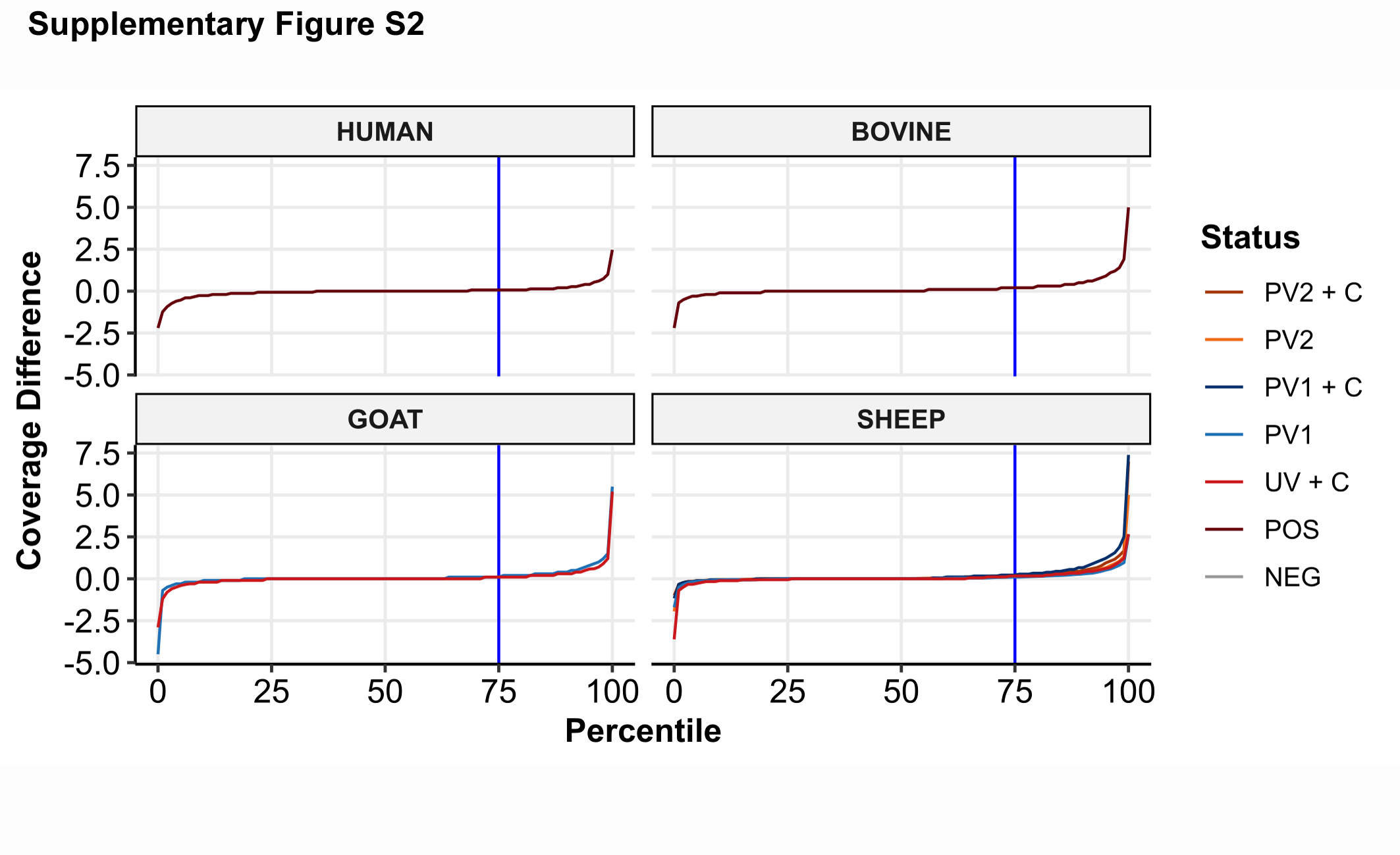

Supplement: Supplementary FIGURE S2 — Method 1 percentiles. Line plots showing the percentiles of differences in coverage values between the positive and negative groups within each species. The percentile values were calculated from 0% to 100% at 1% steps. Coverage is the product between a domain’s response frequency and median response count. POS, C. burnetii positive; PV1, post-C. burnetii phase I vaccination; PV2, post-C. burnetii phase II vaccination; UV, unvaccinated; +C, C. burnetii challenge. [file Image_2.tif]

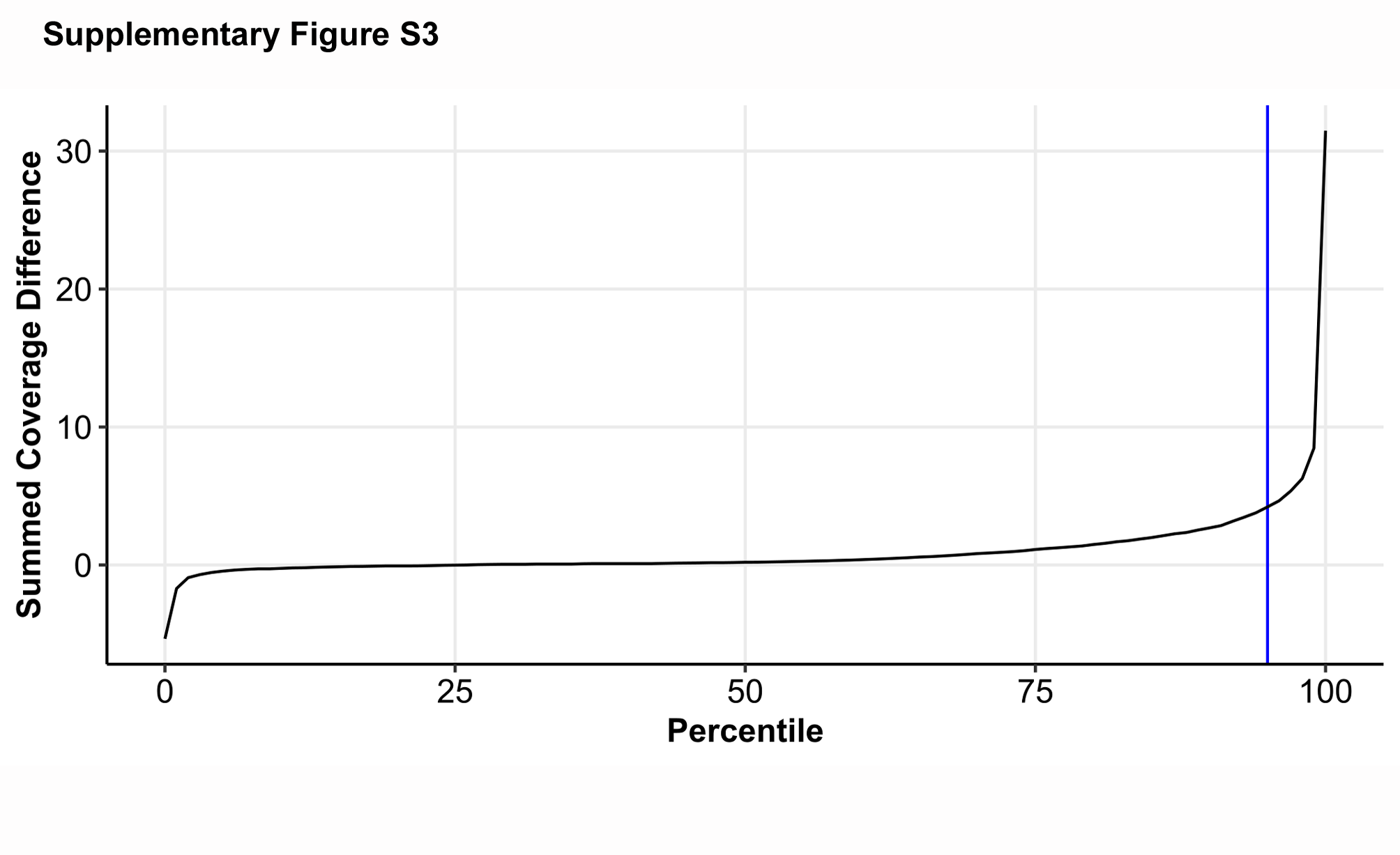

Supplement: Supplementary FIGURE S3 — Method 2 percentiles. Line plot showing the percentiles of the summed differences in coverage values between the positive and negative groups within each species. The percentile values were calculated from 0% to 100% at 1% steps. Coverage is the product between a domain’s response frequency and the median response count. [file Image_3.tif]
