# Supplementary material for: Genome-wide epitope mapping across multiple host species reveals significant diversity in antibody responses to Coxiella burnetii vaccination and infection
Source: Front Immunol. 2023 Oct 26;14:1257722. doi: 10.3389/fimmu.2023.1257722 (PMC10637584; doi:10.3389/fimmu.2023.1257722)
Supplement: Supplementary file 5 [file Table_1.docx]

**Supplementary Table S1: Generalized linear models of pairwise Jaccard similarities between sets of *C. burnetii* protein domains recognized by individual sera**

|  |  | **Comparison** | **Estimate*** | **Std. Error** | **T-statistic** | **P-value** | **N**** |
| --- | --- | --- | --- | --- | --- | --- | --- |
| **Unpaired serum samples** | | | | | | | |
| Degrees of Freedom: |  | No match (intercept) | 0.3266 | 0.0029 | 113.68 | 0.00E+00 | 2030 |
| 5777 Total |  | Both positive | 0.0174 | 0.0041 | 4.22 | 2.53E-05 | 2628 |
| 5772 Residual |  | Both negative | -0.0252 | 0.0071 | -3.55 | 3.84E-04 | 595 |
|  |  | Species match | 0.0206 | 0.0063 | 3.25 | 1.15E-03 | 1438 |
|  |  | Species match & positive | -0.0040 | 0.0085 | -0.47 | 6.35E-01 | 718 |
|  |  | Species match & negative | 0.0404 | 0.0130 | 3.11 | 1.85E-03 | 195 |
| **Paired sheep serum samples** | | | | | | | |
| Degrees of Freedom: |  | No match (intercept) | 0.3036 | 0.0056 | 54.66 | 0.00E+00 | 510 |
| 1127 Total |  | Same Id | 0.2112 | 0.0236 | 8.96 | 1.32E-18 | 42 |
| 1122 Residual |  | Same Status | 0.0027 | 0.0160 | 0.17 | 8.67E-01 | 228 |
|  |  | Both positive | 0.1012 | 0.0087 | 11.60 | 1.83E-29 | 435 |
|  |  | Both negative | -0.0532 | 0.0197 | -2.70 | 7.05E-03 | 153 |
|  |  | Same Id & positive | 0.0000 | 0.0437 | 0.00 | 1.00E+00 | 12 |

*Estimate = “No match (intercept)” indicates background Jaccard similarity when individuals have nothing in common, all other estimate values are relative to intercept.

**N = number of pairwise Jaccard similarities in comparison group.
