# Supplementary material for: Genome-wide epitope mapping across multiple host species reveals significant diversity in antibody responses to Coxiella burnetii vaccination and infection
Source: Front Immunol. 2023 Oct 26;14:1257722. doi: 10.3389/fimmu.2023.1257722 (PMC10637584; doi:10.3389/fimmu.2023.1257722)
Supplement: Supplementary file 7 [file Table_3.docx]

**Supplementary Table S3: Contingency matrix of TMHMM 2.0 and PSORTb 3.0 predicted localizations of vaccine candidate antigen domains**

|  |  | **TMHMM 2.0 localization** | | |  |
| --- | --- | --- | --- | --- | --- |
|  |  | inside | outside | TMhelix* | PSORTb 3.0 total |
| **PSORTb 3.0 localization** | Cytoplasmic | 4 | 273 | 0 | 277 |
|  | Cytoplasmic Membrane | 27 | 62 | 2 | 91 |
|  | Extracellular | 0 | 2 | 0 | 2 |
|  | Outer Membrane | 0 | 5 | 0 | 5 |
|  | Periplasmic | 0 | 4 | 0 | 4 |
|  | Unknown | 18 | 96 | 0 | 114 |
|  | TMHMM 2.0 total | 49 | 442 | 2 |  |

*Transmembrane helix
